# Monte Carlo calculations of PET coincidence timing: single and double-ended readout

**DOI:** 10.1088/0031-9155/60/18/7309

**Published:** 2015-09-09

**Authors:** Stephen E Derenzo, Woon-Seng Choong, William W Moses

**Affiliations:** Life Sciences Division, Lawrence Berkeley National Laboratory, Berkeley, CA, USA

**Keywords:** positron emission tomography, time of flight, scintillator, coincidence resolving time, Monte Carlo, lower bound

## Abstract

We present Monte Carlo computational methods for estimating the coincidence resolving time (CRT) of scintillator detector pairs in positron emission tomography (PET) and present results for Lu_2_SiO_5_ : Ce (LSO), LaBr_3_ : Ce, and a hypothetical ultra-fast scintillator with a 1 ns decay time. The calculations were applied to both single-ended and double-ended photodetector readout with constant-fraction triggering. They explicitly include (1) the intrinsic scintillator properties (luminosity, rise time, decay time, and index of refraction), (2) the exponentially distributed depths of interaction, (3) the optical photon transport efficiency, delay, and time dispersion, (4) the photodetector properties (fill factor, quantum efficiency, transit time jitter, and single electron response), and (5) the determination of the constant fraction trigger level that minimizes the CRT. The calculations for single-ended readout include the delayed photons from the opposite reflective surface. The calculations for double-ended readout include (1) the simple average of the two photodetector trigger times, (2) more accurate estimators of the annihilation photon entrance time using the pulse height ratio to estimate the depth of interaction and correct for annihilation photon, optical photon, and trigger delays, and (3) the statistical lower bound for interactions at the center of the crystal. For time-of-flight (TOF) PET we combine stopping power and TOF information in a figure of merit equal to the sensitivity gain relative to whole-body non-TOF PET using LSO.

For LSO crystals 3 mm × 3 mm × 30 mm, a decay time of 37 ns, a total photoelectron count of 4000, and a photodetector with 0.2 ns full-width at half-maximum (fwhm) timing jitter, single-ended readout has a CRT of 0.16 ns fwhm and double-ended readout has a CRT of 0.111 ns fwhm. For LaBr_3_ : Ce crystals 3 mm × 3 mm × 30 mm, a rise time of 0.2 ns, a decay time of 18 ns, and a total of 7600 photoelectrons the CRT numbers are 0.14 ns and 0.072 ns fwhm, respectively. For a hypothetical ultra-fast scintillator 3 mm × 3 mm × 30 mm, a decay time of 1 ns, and a total of 4000 photoelectrons, the CRT numbers are 0.070 and 0.020 ns fwhm, respectively. Over a range of examples, values for double-ended readout are about 10% larger than the statistical lower bound.

## 1. Introduction

This paper presents Monte Carlo approaches that simulate all the important factors that limit the CRT in PET, including (1) the scintillator rise time, decay time, length, and index of refraction, (2) the distribution of annihilation photon transit times and interaction depths, (3) the distribution of transit times of optical photons, (4) the number and time distribution of the photoelectrons, (5) the timing jitter and single electron response (SER) of the photodetector, (6) optimal constant fraction triggering, and (7) both single-ended and double-ended readout.

It advances previous work in modeling the optical photon dispersion as a function of DOI and provides numerical results for single-ended and double-ended readout for a wide range of situations. For double-ended readout it corrects the constant-fraction trigger times for depth-dependent annihilation photon, optical photon, and trigger delays and then combines them in a statistically weighted average that is only about 10% higher than the statistical lower bound. It shows the conditions necessary for achieving CRT values as low as 0.01 ns fwhm.

One objective of the paper is to provide clear, detailed program steps so that a computer programmer with little knowledge of statistics can write code that computes the CRT for any scintillator rise and decay time, optical photon dispersion, number of photoelectrons, and photodetector SER and time jitter. It is however not a substitute for more accurate calculations that capture the details of the Compton and photoelectric interactions, the transport of the optical photons, and the properties of the photodetector.

The paper is organized as follows. In section 2.1, we describe the history of the different scintillation detectors that have been used in PET. In section 2.2 we summarize previous work that shows how double-ended readout can be used to estimate the depth of interaction (DOI) of an annihilation photon. In section 3 we present our results of Monte Carlo calculations of the optical photon time dispersion and how it can be modeled by a single exponential time decay parameter that is related to the DOI. In section 4 we describe the Monte Carlo calculations used in this work to calculate the CRT for single-ended and double-ended readout, and validate against available measured values. In section 5 we present the results of the calculations for three representative scintillators: LSO, LaBr_3_ : Ce, and a hypothetical ultra-fast scintillator. In section 6 we describe a figure of merit (FOM) for estimating the TOF sensitivity gain and calculate the FOM for the three example scintillators. Section 7 compares five strategies that can be used to estimate the time of arrival of the annihilation photons. Section 8 lists the conclusions from this work. Appendix A lists the variables and abbreviations used. Appendix B describes examples of trigger fraction optimization. Appendix C shows an example of the weighting factors needed for the best statistical estimation of the annihilation photon arrival time. Appendix D presents a numerical method for computing the CRT lower bound and shows that over a range of cases double-ended readout with corrections for the exponential distribution of the DOI gives essentially the same CRT values as when all interactions are at the crystal center and that these CRT values are only about 10% higher than the statistical lower bound.

## 2. Background

In section 2.1 we summarize the history of different scintillators used in PET with special attention to their CRT and TOF PET performance. In section 2.2 we summarize previous work in double-ended readout and the use of pulse height ratios to estimate the DOI.

### 2.1. Scintillation detectors used in PET

Timing resolution has always been an important factor in PET, because events are identified by timing the arrival of pairs of 511 keV annihilation photons and rejecting those whose times are so different that it is unlikely that they could have been emitted by the same positron. The earliest positron tomographs used NaI(Tl), and these provided coincidence windows typically 10 ns wide for the rejection of accidental coincidence events (Rankowitz *et al* 1962, Robertson *et al* 1973, Derenzo *et al* 1979). In the 1980s positron tomographs were built that used ultra-fast scintillators (CsF and BaF_2_) to measure the time of flight of the two annihilation photons with sufficient accuracy to locate the position of annihilation within the patient (Mullani *et al* 1980, Terpogossian *et al* 1981, Moszynski *et al* 1984). The ultra-fast scintillation is due to core-valence emission, where the ionization event ejects electrons from a core band, and electrons from the valence band promptly fill the holes and produce photons if their energy is less than the band gap of the material (Valbis *et al* 1985). Because this process has a maximum luminosity of about 2000 photons MeV^−1^, the CRTs were limited to about 0.4 ns fwhm. After its discovery in 1973 (Weber and Monchamp 1973) PET designers switched to the denser scintillator Bi_4_Ge_3_O_12_ (BGO) (Cho and Farukhi 1977, Derenzo *et al* 1981, 1987). It has a much higher photopeak efficiency than NaI(Tl), CsF, and BaF_2_, but its timing resolution is not adequate for TOF PET.

In 1992 Lu_2_SiO_5_ : Ce (LSO) was discovered (Melcher and Schweitzer 1992), and it and the related compound Lu_2−*x*_Y_*x*_SiO_5_ : Ce (LYSO) are now used in almost all positron tomographs. These scintillators have a fast rise time (Derenzo *et al* 2000), 33–40 ns decay time, about 20 000 photons per 511 keV of ionization, and an initial intensity of 500 photons ns^−1^, prompting research in optimizing its timing resolution for TOF PET (Moszynski *et al* 2006, Choong 2009, Moses *et al* 2010, Lecoq 2012, Auffray *et al* 2013, Gundacker *et al* 2013, Lecoq *et al* 2014).

In parallel the fundamental limits of CRT in PET have been explored analytically and with Monte Carlo calculations (Vinke *et al* 2009, Spanoudaki and Levin 2011, Seifert *et al* 2012a, 2012b, Gundacker *et al* 2013). These previous papers focus on single-ended readout and the deterioration of CRT with increasing crystal length (Gundacker *et al* 2014). More recently Seifert and Schaart experimentally explored double-ended readout and averaged the trigger times of the two photodetectors to partially correct for variations in the DOI (Seifert and Schaart 2015). This paper uses Monte Carlo calculations to show that for a variety of cases double-ended readout and full correction for the depth-dependent annihilation photon, optical photon, and trigger delays gives the same CRT as interactions at the crystal center and essentially eliminates the effects of variations in the DOI.

### 2.2. Use of double-ended readout to estimate the DOI

**Figure 1:**
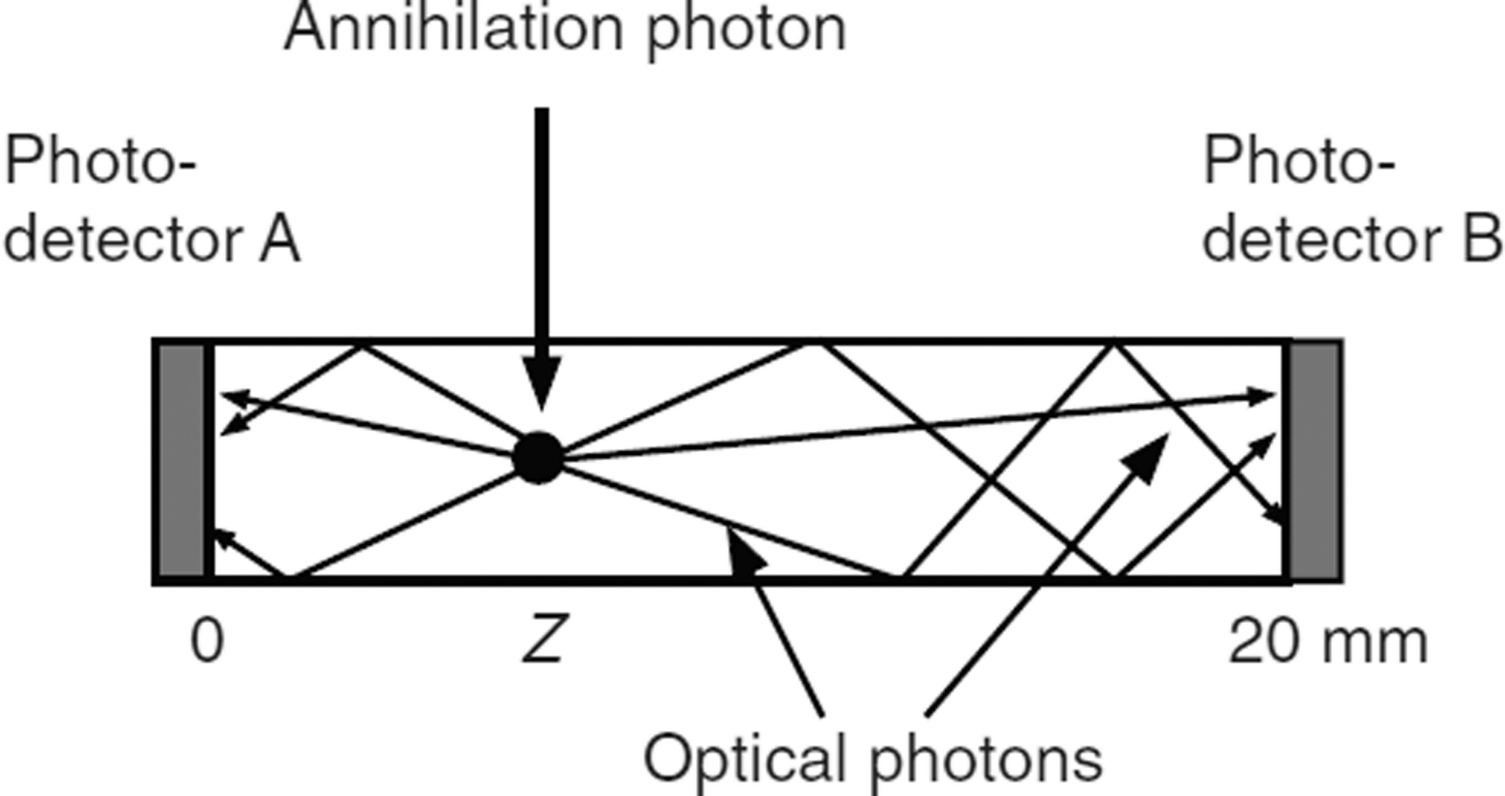
An annihilation photon interacts at depth *Z*, and the depth determines the fraction of the optical photons received by photodetectors A and B.

Yang *et al* (2006) coupled two avalanche photodiodes to opposite surfaces of arrays of 1.5 mm × 1.5 mm × 20 mm long unpolished LSO crystals (figure 1). They used a positron source and an electronically collimated beam to measure the signals in photodetectors A and B as a function of the position of the beam along the crystal. For an interaction point at *Z* = 0, photodetector A received 70% of the photons and photodetector B received 30%. For an interaction point at *Z* = 20 mm, detector B received 70% of the photons and photodetector A received 30%. The percentages were linear functions of the position between those limits.

Generalizing this to a scintillator of length *L* results in the relations:

(1a) $$f_{A}(Z)=a-(2a-1)Z/L$$

(1b) $$f_{B}(Z)=(1-a)+(2a-1)Z/L,$$

where *f*_A_(*Z*) and *f*_B_(*Z*) are the fractions of the signal in photodetectors A and B, respectively, as a function of the DOI *Z*, and *L* is the full length of the scintillator.

The value of *a* = 0.7 is used in later sections because it is within the range that can be can be realized by varying the surface treatment (Yang *et al* 2006, Seifert and Schaart 2015) and is a good compromise between no signal at the distant end (i.e. the *a* = 1 limit) and zero DOI sensitivity (i.e. the *a* = 0.5 limit). In the case where only one photodetector is used and the opposite end surface of the scintillator is reflecting, the relationships determine the fraction that reach the photodetector and the fraction that reach the opposite end and are reflected back. In the case where photodetectors are coupled to both end surfaces of the scintillator, the relations determine the fractions received by the two photodetectors.

## 3. Monte Carlo calculations of the optical photon time dispersion as a function of interaction depth

In previous publications we presented Monte Carlo calculations of the optical photon time dispersion and its characterization as a distribution with a sharp rise (<10 ps) at the time of arrival of the earliest possible photon followed by an exponential decay (Derenzo *et al* 2014) for both rough and polished surfaces (Moses *et al* 2014) followed by a similar pulse of photons reflected from the opposite end. This is consistent with previously published calculations (Yeom *et al* 2013, Gundacker *et al* 2014, Vinke *et al* 2014) and experimental measurements of rectangular LSO crystals (de Haas *et al* 2014).

**Figure 2:**
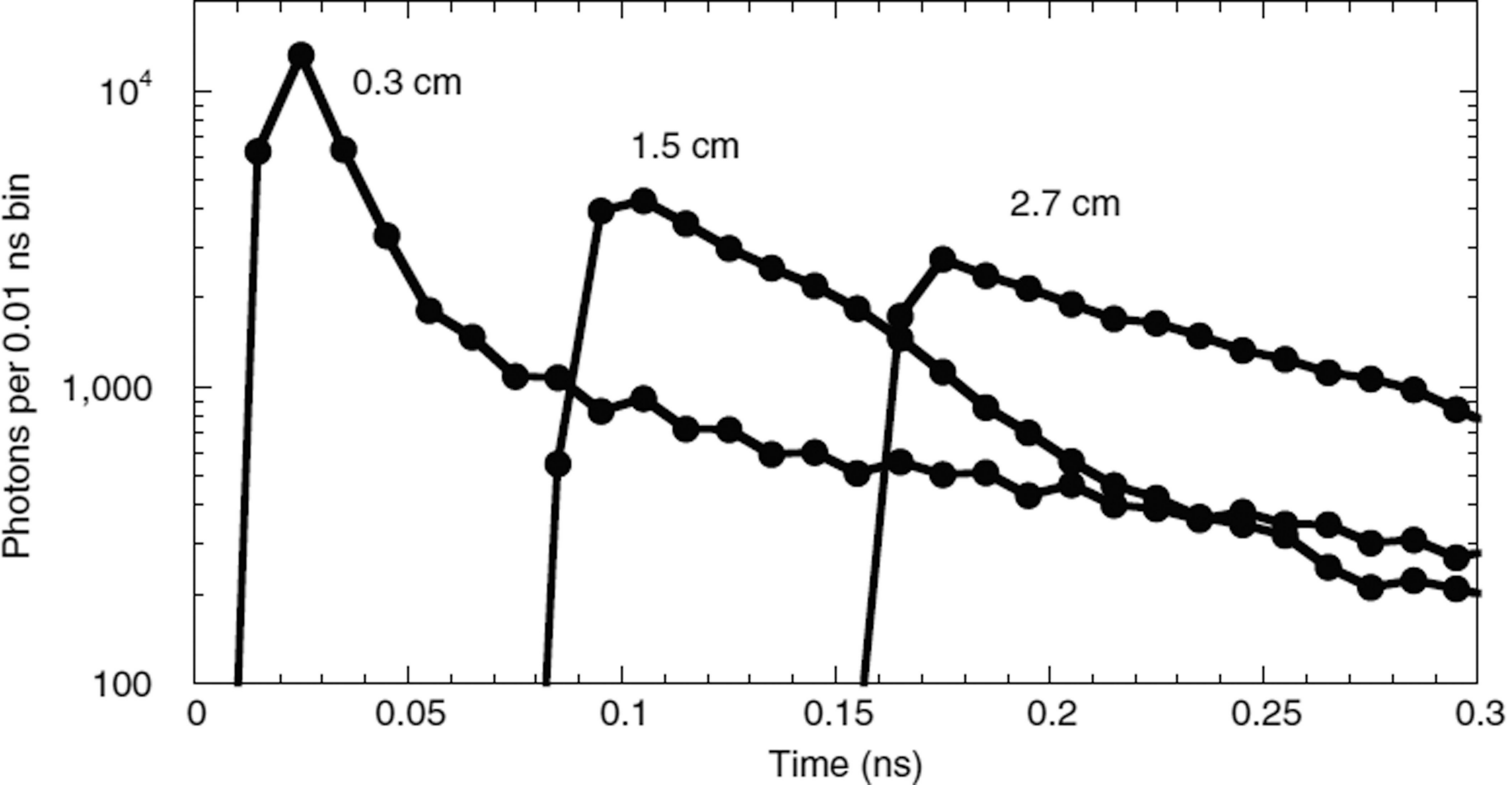
Monte Carlo calculated optical photon time dispersions at distances of 3, 15, and 27 mm from a photodetector coupled to one end surface of a 3 mm × 3 mm × 30 mm LSO crystal with polished surfaces and Teflon external reflector. The other end surface was absorptive to simulate a second photodetector. Lines are provided to guide the eye.

In this section we present Monte Carlo calculations of the optical photon time dispersion as a function of the DOI for a polished 3 mm × 3 mm × 30 mm LSO crystal with an external Teflon reflector using Geant4 (Agostinelli *et al* 2003). Figure 2 shows the time distributions for emission points at distances of 3, 15, and 27 mm from the photodetector. The other end surface was absorptive to simulate a second photodetector. The distribution includes all the optical photons produced in the interaction that reach one end of the crystal including those that are reflected multiple times by the side surfaces. The distributions are well described by the expression exp[−(*T* − *T*_0_)/*D*(*Z*)] where *T*_0_ is the time of arrival of the first possible photon, *D*(*Z*) is a time dispersion parameter, and *Z* is the DOI.

**Figure 3:**
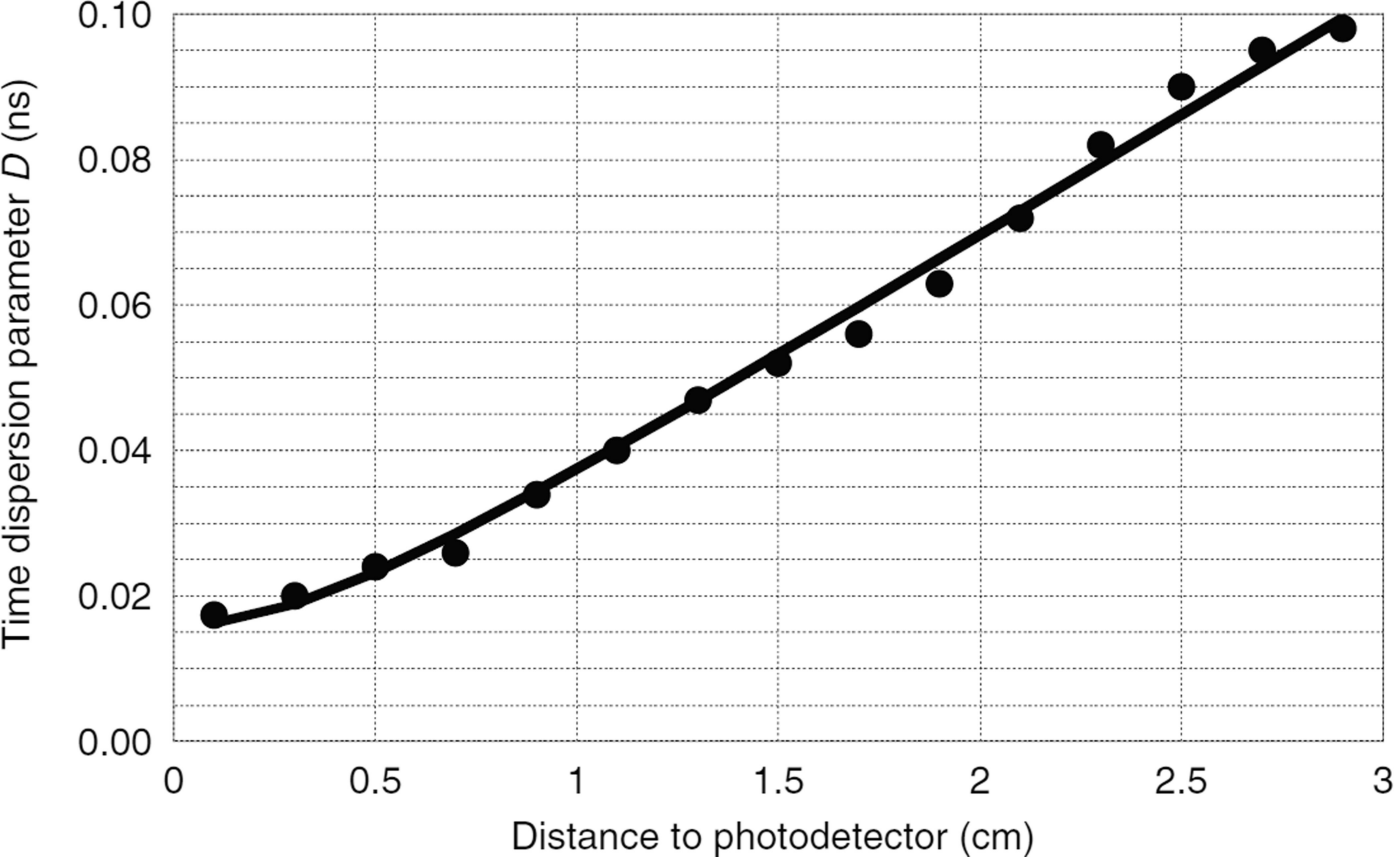
Exponential time dispersion parameter for optical photons as a function of the distance from the interaction point to a photodetector mounted at one end surface of a 3 mm × 3 mm × 30 mm LSO crystal with polished surfaces and Teflon external reflector. The other end surface was absorptive to simulate a second photodetector. Solid line is the best-fit model (equations (2*a*) and (2*b*)).

This calculation was repeated for 15 different values of *Z* along the crystal and the time dispersion parameters are plotted in figure 3. Since the time scale of the time dispersion process depends on the speed of the optical photons in the scintillator, the time dispersion parameter should be proportional to the index of refraction *n*. For example, when using an external specular reflector, increasing the index of refraction from 1.82 (LSO) to 2.1 (LaBr_3_) will have little effect on the paths that the optical photons take but will increase their transit time by the ratio of the refractive indexes. This leads to the following equation for the time dispersion parameters for photons arriving at surfaces A and B

(2a) $$D_{A}(Z)=n\sqrt{d_{1}^{2}+d_{2}^{2}Z^{2}}$$

(2b) $$D_{B}(Z)=n\sqrt{d_{1}^{2}+d_{2}^{2}{\left(L-Z\right)}^{2}},$$

where the optical photon intensity at time *t* after the earliest possible photon is proportional to exp(−*t*/*D*(*Z*)). Fitting these equations to the LSO data of figure 3 gives best-fit values *d*_1_ = 0.008 73 ns and *d*_2_ = 0.0186 ns cm^−1^.

Single-ended readout has the complication that the photons arrive at the photodetector in two swarms, the first from those that travel directly from the interaction point to the photodetector and a delayed swarm from the opposite reflective surface. This is shown in figure 1 of Moses *et al* (2014), figure 11 of Yeom *et al* (2013), figure 4 of de Haas *et al* (2014), figure 18 of Gundacker *et al* (2014), and figure 9 of Vinke *et al* (2014).

## 4. Monte Carlo calculations

In a previous publication we presented the results of 820 Monte Carlo calculations of scintillation detector timing precision that spanned a range of scintillator rise and decay times, numbers of photoelectrons, optical photon time dispersion parameters, and photodetector timing jitters (Derenzo *et al* 2014). In each case the timing precision was calculated for a range of leading edge trigger levels to find the optimum. One important conclusion is that the optimal trigger level is proportional to the number of photoelectrons per ns decay time and that the best strategy is to trigger at a constant fraction of the pulse height. In this work we apply those techniques to PET, where each 511 keV annihilation photon interacts at an exponentially distributed random depth, and where the number of photoelectrons (equations (1*a*) and (1*b*)) and the optical photon time dispersion parameter (equations (2*a*) and (2*b*)) depend on the DOI.

The Monte Carlo calculations are described in four sections. Section 4.1 describes the generation of annihilation photon interaction depths, the fraction of photons that reach the opposite end surfaces of the scintillator, and the number of photoelectrons. Section 4.2 treats the case where one photodetector is attached to the entrance surface A and the rear surface B is reflective. Section 4.3 treats the case where one photodetector is attached to the rear surface B and the entrance surface A is reflective. Section 4.4 treats the case where photodetectors are attached to both surfaces A and B, and their signals are used to estimate the DOI, correct for annihilation photon, optical photon, and trigger delays, and provide the best statistical estimate of the time when the annihilation photon entered the scintillator. In almost all cases 100 000 interaction events were used in the calculations. If *N*_g_ is a large number of interaction events, the standard error in the standard deviation σ of the distribution of entrance times is σ/(2*N*_g_)^1/2^ (Olive *et al* 2014). Since the CRT is a multiple of the standard deviation, the standard error in the CRT is CRT/(2*N*_g_)^1/2^, which for *N*_g_ = 100 000 is about CRT/400. In appendix A table A1 lists the variables used in the calculations, and table A2 lists the abbreviations used in the text.

### 4.1. Interaction, emission, and photoelectron production processes

This section describes the generation of annihilation photon interaction depths, the fraction of photons that reach the opposite end surfaces of the scintillator, and the number of photoelectrons plus a Gaussian noise term that has a mean equal to zero and a variance equal to the expected number of photoelectrons. These photoelectron counts will be used in sections 4.2 and 4.3 for single-ended readout and in section 4.4 for double-ended readout.

#### 4.1.1

For each absorbed annihilation photon *k*, select a random number *R_k_* from a set of numbers uniformly distributed between exp(−*L*/μ) and 1. Compute the interaction depth *Z_k_* = −μ ln(*R_k_*) in the scintillator, where μ is the exponential attenuation depth of the annihilation photons. *Z* = 0 at the entrance surface A, and *Z* = *L* at the rear surface B.

#### 4.1.2

For each interaction at depth *Z_k_*, compute the expected number of photoelectrons *N*_A*k*_ and *N*_B*k*_ that are produced by the photons that will reach end surfaces A and B, respectively (equations (1*a*) and (1*b*)). *N*_pe_ is the product of the scintillator luminosity (photons MeV^−1^), the energy deposited (511 keV), the photon transport efficiency, the photodetector fill factor, and the photodetector quantum efficiency.

(3a) $$N_{Ak}=N_{pe}f_{A}(Z_{K})$$

(3b) $$N_{Bk}=N_{pe}f_{B}(Z_{K}).$$

#### 4.1.3

Compute the number of observed photoelectrons *m*_A*k*_ and *m*_B*k*_ by adding to *N*_A*k*_ and *N*_B*k*_ random numbers selected from a set with a Gaussian distribution, mean 0, and variance *N*_A*k*_ and *N*_B*k*_, respectively.

(4a) $$m_{Ak}=N_{Ak}+\text{Gaussian }(\text{mean}=0,\text{ variance}=N_{Ak})$$

(4b) $$m_{Bk}=N_{Bk}+\text{Gaussian }(\text{mean}=0,\text{ variance}=N_{Bk}).$$

### 4.2. The Monte Carlo calculation for single-ended readout using one photodetector at entrance surface A

**Figure 4:**
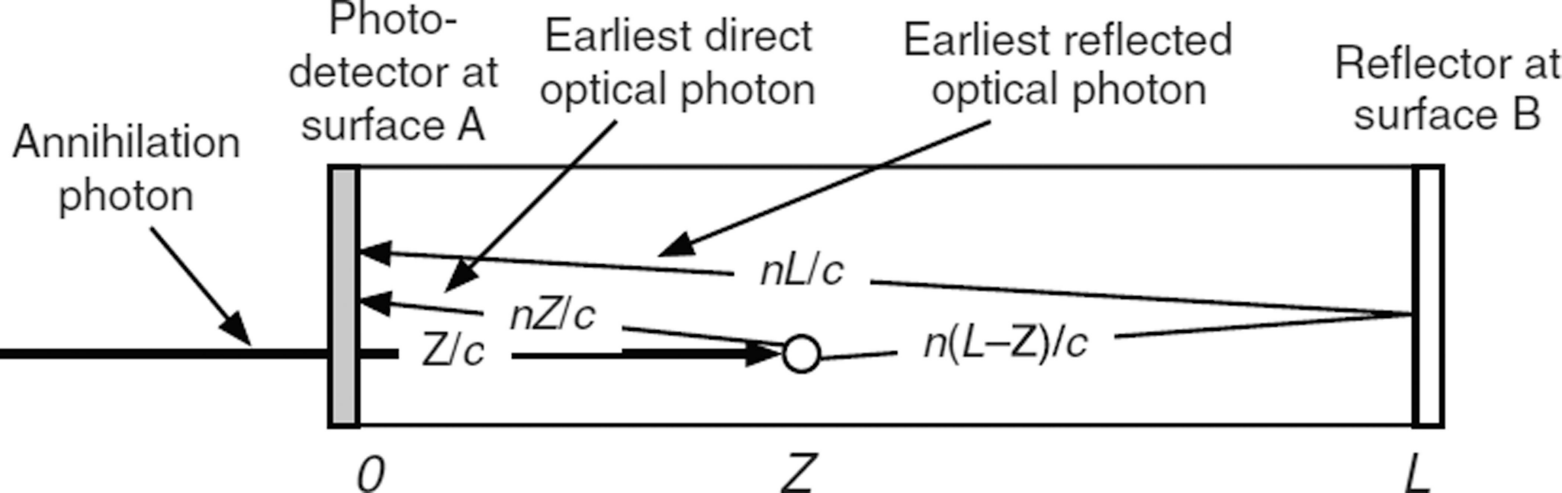
An annihilation photon enters the scintillator at surface A at time 0 and interacts at a depth *Z* at time *Z*/*c*. The earliest possible direct optical photon reaches photodetector A at time *Z*/*c* + *nZ*/*c*. The earliest possible optical photon reflected from surface B reaches photodetector A at time *Z*/*c* + *n*(2*L* − *Z*)/*c*.

**Figure 5:**
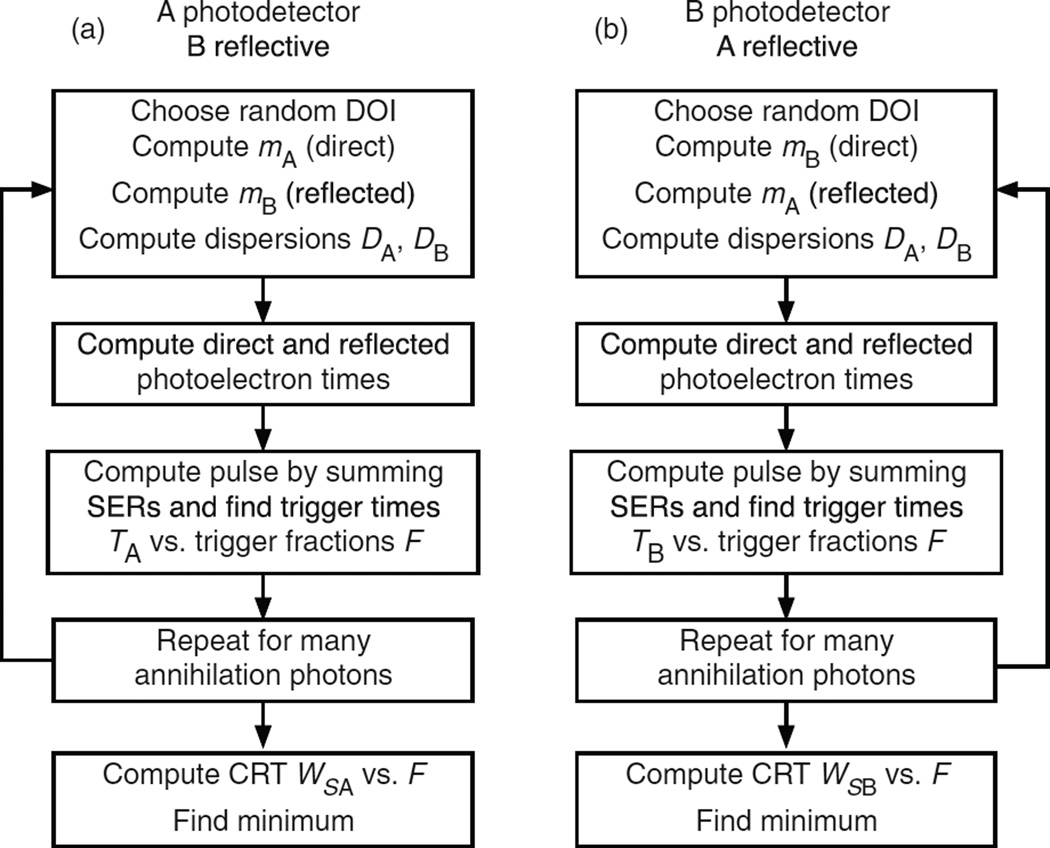
Simplified diagram of the Monte Carlo calculation steps for two modes of single-ended readout. See sections 4.1–4.3 for details.

In this case the entrance surface A is coupled to a photodetector, and the rear surface B is reflecting (figure 4). After each interaction photodetector A receives an initial swarm of photons and a delayed swarm of photons reflected from rear surface B. The Monte Carlo code generates photoelectron times based on the two swarms, computes the pulse amplitude by summing the SER pulses, and determines constant-fraction trigger times for a full range of trigger levels. This is shown as a simplified block diagram in figure 5(a) and is described in detail in the following sections.

#### 4.2.1

Tabulate the photodetector SER *S*(*t*) as a bi-exponential with rise time *S*_r_ and decay time *S*_d_ on a fine time grid (0.0001 ns was used in this work).

(5) $$S(t)=[\text{exp}(-t/S_{d})-\text{exp}(-t/S_{r})]/(S_{d}-S_{r}).$$

Define a table *F*_n_ of trigger fractions from 0 to 1 (see figure B1 for examples of those used in this work).

#### 4.2.2

For each interacting annihilation photon *k*, use steps 4.1.1 to 4.1.3 compute the DOI *Z_k_*, the number *m*_Ak_ of photoelectrons from photons that reach the entrance surface A directly, and the number *m*_Bk_ of photoelectrons from photons that are reflected from the rear surface B.

#### 4.2.3

Compute the optical photon time dispersion parameters *D*_Ak_ for photons that reach photodetector A directly (minimum path length *Z*_k_) and *D*_Bk_ for the photons that are reflected from rear surface B (minimum path length 2*L* − *Z_k_*). See section 3 for a discussion of these equations and the determination of the parameters *d*_1_ and *d*_2_.

(6a) $$D_{Ak}=n\sqrt{d_{1}^{2}+d_{2}^{2}Z_{k}^{2}}$$

(6b) $$D_{Bk}=n\sqrt{d_{1}^{2}+d_{2}^{2}{\left(2L-Z_{k}\right)}^{2}}.$$

#### 4.2.4

After the arrival of annihilation photon *k* at entrance surface A, the earliest possible photon will reach photodetector A at time *Z_k_*/*c* + *nZ_k_*/*c*, and the earliest possible photon reflected from rear surface B will reach photodetector A at time *Z_k_*/*c* + *n*(2*L* − *Z_k_*)/*c*. Select *R*_1_, *R*_2_ ,…, *R*_3*m*_A*k*_+3*m*_B*k*__ from a set of random numbers uniformly distributed between 0 and 1. The times of the direct *m*_Ak_ photoelectrons (*m* = 1 to *m*_Ak_) are

$$T_{m}=Z_{k}/c+nZ_{k}/c-\tau_{r}\text{ ln}(R_{3m-2})-\tau_{d}\text{ ln}(R_{3m-1})-D_{Ak}\text{ ln}(R_{3m}).$$

The times of the *m*_Bk_ delayed photoelectrons (*m* = *m*_Ak_ + 1 to *m*_Ak_ + *m*_Bk_) are

$$T_{m}=Z_{k}/c+n(2L-Z_{k})/c-\tau_{r}\text{ ln}(R_{3m-2})-\tau_{d}\text{ ln}(R_{3m-1})-D_{Bk}\text{ ln}(R_{3m}).$$

#### 4.2.5

To each time *T_m_* add a random time with a Gaussian distribution (mean 0, fwhm *J*) to simulate the time jitter of the photodetector. There is an additional delay from the arrival of the photon at the photodetector and the mean of the Gaussian distribution, but this is the same for all photons, does not contribute to the CRT, and is set to zero.

Note that steps 4.2.4 and 4.2.5 include the annihilation photon transit time, the scintillator rise and decay time, the optical photon time delay and dispersion, and the photodetector timing jitter.

#### 4.2.6

Sort all photoelectron times *T_m_, m* = 1 to *m*_A*k*_ + *m*_B*k*_.

#### 4.2.7

Find the maximum photodetector output pulse amplitude *P*_A*k*_(max) and use iterative linear interpolation to solve the equation *P*(*t*)/*P*_A*k*_(max) = *F_n_* to determine the trigger time *t* = *T*_A*kn*_ for each fractional trigger level *F_n_*. *P*(*t*) sums the SER amplitudes (computed in step 4.2.1) from all photoelectrons whose times are less than *t*. Sorting the times in step 4.2.6 allows the summation to be concluded at the first *T_m_* > *t*.

(7) $$P(t)=\sum_{m=1}^{m_{A}+m_{B}}S(t-T_{m})\text{ for }t>T_{m}.$$

#### 4.2.8

Repeat steps 4.2.2 to 4.2.7 for *k* = 1 to *N_g_* interactions and compute the standard deviation of the trigger times *T*_*A*kn_ for each fractional trigger level *F_n_*. Multiply by 2.3548 to convert to the single detector fwhm. Multiply by 1.4142 to compute the CRT *W*_SA_(*F_n_*) for a pair of detectors with single-sided readout of surface A.

#### 4.2.9

Find the optimal trigger fraction *F*_opt_ and CRT *W*_SA_.

### 4.3. The Monte Carlo calculation for single-ended readout using one photodetector at rear surface B

**Figure 6:**
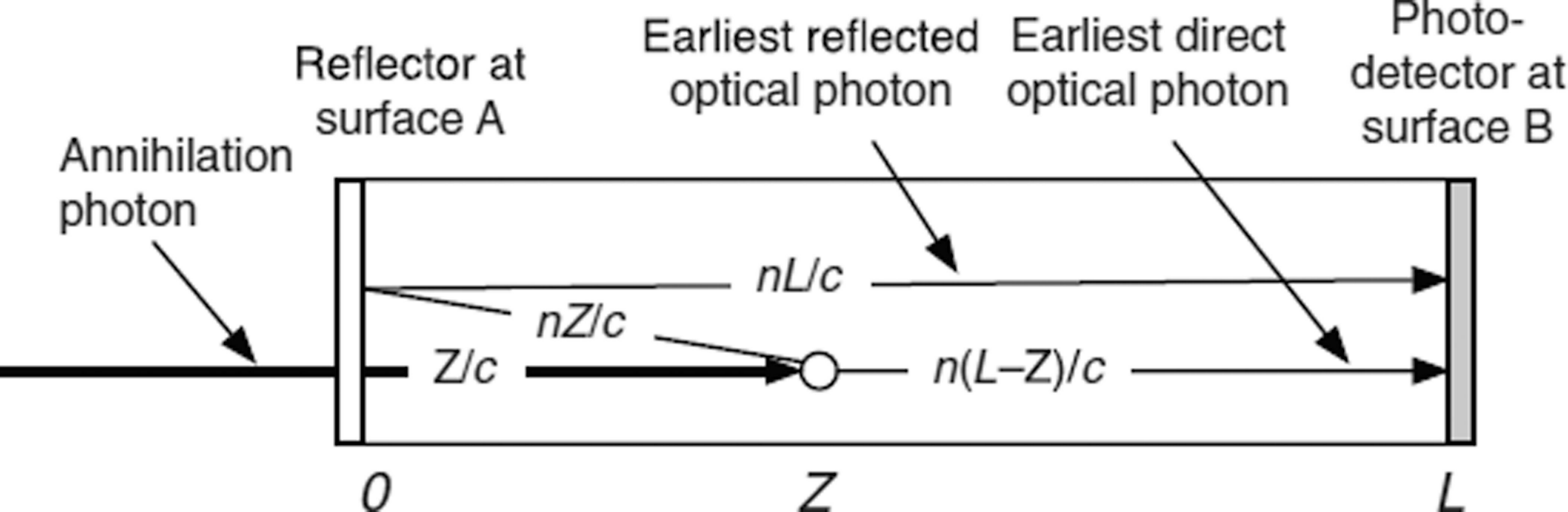
An annihilation photon enters the scintillator at surface A at time 0 and interacts at a depth *Z* at time *Z*/*c*. The earliest possible optical photon reaches photodetector B at time *Z*/*c* + *n*(*L* − *Z*)/*c*. The earliest possible optical photon reflected from surface A reaches photodetector B at time *Z*/*c* + *nZ/c* + *nL*/*c*.

In this case the rear surface B is coupled to the photodetector, and the entrance surface A is reflecting (figure 6). After each interaction photodetector B receives an initial swarm of photons and a delayed swarm of photons reflected from the entrance surface A. The Monte Carlo code generates photoelectron times based on the two swarms, computes the pulse amplitude by summing the SER pulses, and determines constant-fraction trigger times for a full range of trigger levels. This is shown as a simplified block diagram in the right side of figure 5(b) and is described in detail in the following sections.

#### 4.3.1

Tabulate the SER on a fine time grid and define a table of trigger fractions from 0 to 1 (as in section 4.2.1).

#### 4.3.2

For each interacting annihilation photon *k*, use steps 4.1.1 to 4.1.3 compute the DOI *Z_k_*, the number *m*_Bk_ of photoelectrons from photons that reach end surface B directly, and the number *m*_Ak_ of photoelectrons from photons that are reflected from entrance surface A.

#### 4.3.3

Compute the optical photon time dispersion parameters *D*_B*k*_ for photons that reach photodetector B directly (minimum path length *L* − *Z_k_*) and *D*_A*k*_ for the photons that are reflected from entrance surface A (minimum path length *L* + *Z_k_*).

(8a) $$D_{Bk}=n\sqrt{d_{1}^{2}+d_{2}^{2}{\left(L-Z_{k}\right)}^{2}}$$

(8b) $$D_{Ak}=n\sqrt{d_{1}^{2}+d_{2}^{2}{\left(L+Z_{k}\right)}^{2}}.$$

#### 4.3.4

After the arrival of annihilation photon *k* at entrance surface A, the earliest possible photon will reach photodetector B at time *Z*_k_/*c* + *n*(*L* − *Z_k_*)/*c*, and the earliest possible photon reflected from surface A will reach photodetector B at time Z_k_/c + *n*(*L* + *Z_k_*)/c. Select *R*_1_, *R*_2_, …, *R*_3*m*_A*k*_+3*m*_B*k*__ from a set of random numbers uniformly distributed between 0 and 1. The times of the direct *m*_Bk_ photoelectrons (*m* = 1 to *m*_Bk_) are

$$T_{m}=Z_{k}/c+n(L-Z_{k})/c-\tau_{r}\text{ ln}(R_{3m-2})-\tau_{d}\text{ ln}(R_{3m-1})-D_{Ak}\text{ ln}(R_{3m}).$$

The times of the *m*_Ak_ delayed photoelectrons (*m* = *m*_B*k*_ + 1 to *m*_B*k*_ + *m*_A*k*_) are

$$T_{m}=Z_{k}/c+n(L+Z_{k})/c-\tau_{r}\text{ ln}(R_{3m-2})-\tau_{d}\text{ ln}(R_{3m-1})-D_{Bk}\text{ ln}(R_{3m}).$$

Use steps 4.2.5 to 4.2.9 to compute the maximum pulse amplitude *P*_B*k*_(max), the trigger times *T*_Bk*n*_ for each *F_n_*, and the optimal CRT *W*_SB_ for the single-sided readout of surface B.

### 4.4. The Monte Carlo calculations for double-ended readout

**Figure 7:**
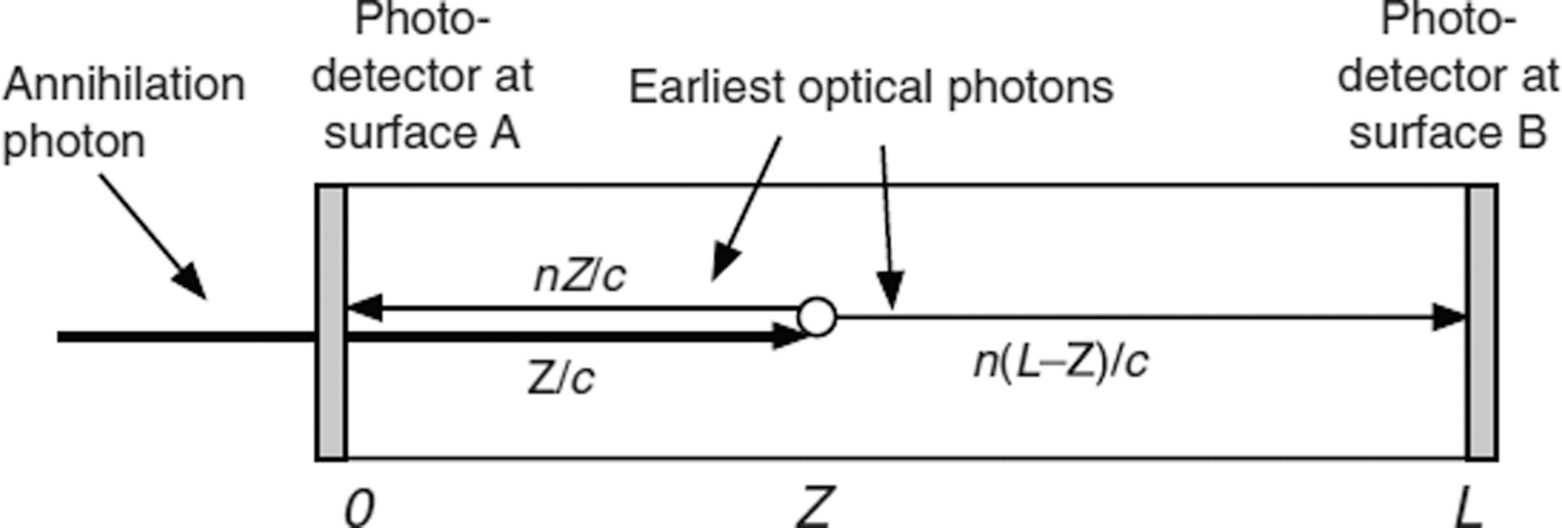
An annihilation photon enters the scintillator at surface A at time 0 and interacts at a depth *Z* at time *Z*/*c*. The earliest possible optical photon reaches photodetector A at time *Z*/*c* + *nZ*/*c*. The earliest possible optical photon reaches photodetector B at time *Z*/*c* + *n*(*L* − *Z*)/*c*.

**Figure 8:**
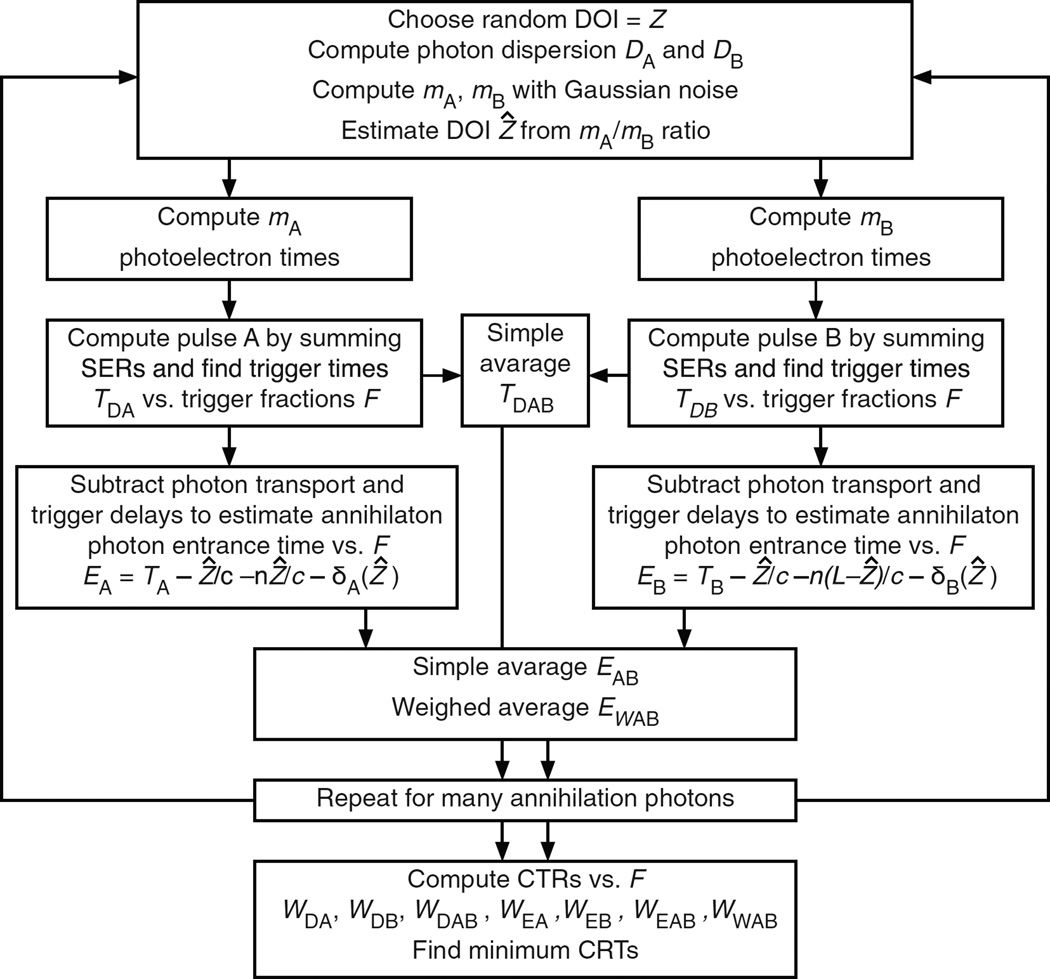
Simplified diagram of the Monte Carlo calculation steps for double-ended readout. See sections 4.4.1 to 4.4.15 for details.

In this case both surfaces A and B are coupled to photodetectors (figure 7). The pulse height ratio is used to estimate the DOI, and both photodetector trigger times are corrected for the depth-dependent annihilation photon, optical photon, and trigger delays to provide an estimator for the time that the annihilation photon entered surface A. This is shown as a simplified block diagram in figure 8 and is described in detail in the following sections.

#### 4.4.1

Tabulate the SER on a fine time grid and define a table of trigger fractions from 0 to 1 (as in section 4.2.1).

#### 4.4.2

For each interacting annihilation photon *k*, use steps 4.1.1 to 4.1.3 compute the DOI *Z_k_*, the number *m*_Ak_ of photoelectrons from photons that reach photodetector A, and the number *m*_Bk_ of photoelectrons from photons that reach photodetector B.

#### 4.4.3

Compute the optical photon time dispersion parameters *D*_Ak_ for the photons that reach photodetector A (minimum path length *Z_k_*) and *D*_Bk_ for the photons that reach photodetector B (minimum path length *L* − *Z_k_*)

(9a) $$D_{Ak}=n\sqrt{d_{1}^{2}+d_{2}^{2}Z_{k}^{2}}$$

(9b) $$D_{Bk}=n\sqrt{d_{1}^{2}+d_{2}^{2}{\left(L-Z_{k}\right)}^{2}}.$$

#### 4.4.4

After the arrival of annihilation photon *k* at entrance surface A, the earliest possible photon at photodetector A will arrive at time *Z_k_*/*c* + *nZ_k_*/*c*, and the earliest possible photon at photodetector B will arrive at time *Z_k_*/*c* + *n*(*L* − *Z*_k_)/*c*. Select *R*_1_, *R*_2_, …, *R*_3*m*_A*k*_+3*m*_B*k*__ from a set of random numbers uniformly distributed between 0 and 1. The times of the *m*_Ak_ photoelectrons (*m* = 1 to *m*_Ak_) in photodetector A are:

$$T_{m}=Z_{k}/c+nZ_{k}/c-\tau_{r}\text{ ln}(R_{3m-2})-\tau_{d}\text{ ln}(R_{3m-1})-D_{Ak}\text{ ln}(R_{3m}).$$

The times of the *m*_Bk_ photoelectrons (*m* = *m*_Ak_ + 1 to *m*_Ak_ + *m*_Bk_) in photodetector B are:

$$T_{m}=Z_{k}/c+n(L-Z_{k})/c-\tau_{r}\text{ ln}(R_{3m-2})-\tau_{d}\text{ ln}(R_{3m-1})-D_{Bk}\text{ ln}(R_{3m}).$$

#### 4.4.5

To each photoelectron time *T_m_* add a random time with a Gaussian distribution, mean 0, fwhm *J* to simulate the time jitter of the photodetector. There is an additional delay from the arrival of the photon at the photodetector and the mean of the Gaussian distribution, but this is the same for all photons, does not contribute to the CRT, and is set to zero.

Note that steps 4.4.4 and 4.4.5 include the annihilation photon transit time, the scintillator rise and decay time, the optical photon time delay and dispersion, and the photodetector timing jitter.

#### 4.4.6

Sort all photoelectron times *T_m_, m* = 1 to *m*_Ak_ + *m*_Bk_.

#### 4.4.7

Find the maximum photodetector output pulse amplitudes *P*_A*k*_(max) and *P*_B*k*_(max), and use iterative linear interpolation to solve the equations *P*(*t*)/*P*_A*k*_(max) = *F_n_* and *P*(*t*)/*P*_B*k*_(max) = *F*_n_ to determine the trigger times *T*_A*kn*_ and *T*_B*kn*_ for the two photodetectors A and B for each fractional trigger level *F_n_*. See section 4.2.7 for the computation of *P*(*t*).

#### 4.4.8

Compute the simple average *T*_AB*kn*_ = (*T*_A*kn*_ + *T*_B*kn*_)/2, which corrects for the optical photon transit time but not the depth-dependent annihilation photon transit time or the trigger delays. It can be seen from step 4.4.4 that a variation Δ*Z* in the DOI causes the photodetector A photoelectron times to vary as Δ*Z*/*c* + *n*Δ*Z*/*c* and the photodetector B photoelectron times to vary as Δ*Z*/*c* − *n*Δ*Z*/*c*. The simple average of the photoelectron times causes the trigger time to vary as Δ*Z*/*c*, and its accuracy is limited by variations in the annihilation photon interaction depth.

The following steps estimate the DOI and correct the constant-fraction trigger times for the depth-dependent annihilation photon, optical photon, and trigger delays. The steps then compute an inverse-variance weighted average of the corrected photodetector times to estimate the time that the annihilation photon entered surface A. A similar process would occur in the electronics of a positron tomograph that uses double-ended readout and digital processing.

#### 4.4.9

Estimate the DOI *Ẑ*, using the functional inverse of equations (1*a*) and (1*b*).

(10) $${Ẑ}_{k}=(L/2)[1-(m_{Ak}-m_{Bk})/(m_{Ak}+m_{Bk})/(2a-1)].$$

In a PET system the pulse heights could be used.

$${Ẑ}_{k}=(L/2)[1-(P_{Ak}(\text{max})-P_{Bk}(\text{max}))/(P_{Ak}(\text{max})+P_{Bk}(\text{max}))/(2a-1)].$$

#### 4.4.10

Correct the trigger times *T*_A*kn*_ and *T*_B*kn*_ for the annihilation photon, optical photon, and trigger delays (see note 1 below) to estimate the entrance times *E*_A*kn*_ and *E*_B*kn*_ for the tabulated values of the trigger fractions *F_n_*.

(11a) $$E_{Akn}=T_{Akn}-{Ẑ}_{k}/c-n{Ẑ}_{k}/c-\delta_{Akn}$$

(11b) $$E_{Bkn}=T_{Bkn}-{Ẑ}_{k}/c-n(L-{Ẑ}_{k})/c-\delta_{Bkn}.$$

#### 4.4.11

*E*_A*kn*_ and *E*_B*kn*_ are separate estimates of the annihilation photon entrance time that would be zero in the limit of infinite statistics. A simple average is given by

(12) $$E_{ABkn}=(E_{Akn}+E_{Bkn})/2.$$

In almost all cases *E*_A*kn*_ has a lower variance (i.e. is more accurate) than *E*_B*kn*_ (see appendix C), and it is better to use the average weighted by the inverse of their variances (see appendix B).

#### 4.4.12

Estimate the inverse variance weighted average of the estimates of the entry times (see note 2 below)

(13) $$E_{WABkn}=(E_{Akn}/V_{Akn}+E_{Bkn}/V_{Bkn})/(1/V_{Akn}+1/V_{Bkn}).$$

#### 4.4.13

Repeat steps 4.4.2 to 4.4.12 for *k* = 1 to *N*_g_ annihilation photons, and tabulate the following quantities as a function of the tabulated trigger fractions (*F_n_*) as in section 4.2.8:

- *W*_DAB_(*F_n_*), the CRT using the simple average of the uncorrected trigger times *T*_A*kn*_ and *T*_B*kn*_.
- *W*_EA_ *and W*_EB_, the CRT using the corrected trigger times E_A*kn*_ and E_B*kn*_, respectively.
- The trigger delays δ_A*jn*_ and δ_B*jn*_, averaged over bands of DOI *Z_j_*.
- *V*_A*jn*_ and *V*_B*jn*_, the variances of the corrected trigger times E_A*kn*_ and E_B*kn*_, respectively, averaged over bands of DOI *Z_j_*.
- *W*_EAB_(*F_n_*), the CRT using the simple average of the depth-dependent corrected trigger times *E*_A*kn*_ and *E*_B*kn*_.
- *W*_WAB_(*F_n_*), the CRT using the inverse variance weighted average of E_A*kn*_ and *E*_B*kn*_.

#### 4.4.14

Find the fractional trigger value *F*_opt_ that minimizes each CRT.

#### 4.4.15

Compute *W*_Z_, the fwhm of the difference between *Z*_k_ and *Ẑ_k_* for *k* = 1 to *N*_g_

Note 1: To perform step 4.4.10 it was necessary to first do a preliminary run of steps 4.4.1 to 4.4.6 for many annihilation photons and tabulate the average trigger delays δ_A*jn*_ and δ_B*jn*_. The trigger delay depends on the DOI *Z_j_*, because the shape of the photodetector pulse depends on the optical photon time dispersion.

Note 2: To perform step 4.4.12 it was necessary to first do a preliminary run of steps 4.4.1 to 4.4.8 and tabulate the variances of *E*_A*kn*_ and *E*_B*kn*_ in bands of *Z_j_* and trigger fraction *F_n_*. This could be done for a PET system by scanning an ultra-fast laser along the length of a component scintillator (as in (de Haas *et al* 2014)), where the laser intensity was adjusted to produce the same number of fluorescent photons as the scintillation photons from an annihilation photon interaction.

### 4.5. Comparison between the Monte Carlo calculations and measured values

Seifert *et al* measured the CRT for opposing pairs of 0.5 cm long crystals of LSO and LaBr_3_ : Ce coupled on one end to SiPM photodetectors, and they also calculated the statistical lower bound (Seifert *et al* 2012b). Table 1 lists the model input parameters and compares them with the single-ended readout Monte Carlo calculations described in section 4.3. The agreement among all three values is within 2% for both LSO and LaBr_3_ : Ce.

In a later publication Seifert *et al* reported the CRT for opposing pairs of 3 mm × 3 mm × 20 mm crystals of LSO (0.2% Ca, τ_d_ = 33 ns) where both ends were coupled to SiPM photodetectors (Seifert and Schaart 2015). With uniform side illumination they averaged the trigger times of both photodetectors, subtracted in quadrature the time resolution of the trigger detector, and multiplied by 1.4142 to convert to the CRT. They obtained a CRT of 0.180 ns fwhm for etched crystals (*N*_pe_ = 4000) and 0.174 ns fwhm for polished crystals (*N*_pe_ = 3600). The Monte Carlo code described in section 4.4 calculated a CRT *W*_DAB_ of 0.157 ns fwhm, assuming *N*_pe_ = 3800, τ_r_ = 0.09 ns, τ_d_ = 33 ns, and *J* = 0.3 ns fwhm. The disagreement (about 12%) is minor and most likely due to the imperfect characterization of experimental factors.

## 5. Results of Monte Carlo calculations of CRT

In the following sections we first describe three scintillators and a high-performance photodetector and then use the Monte Carlo procedures described in section 4 to compute the CRT values that can be achieved in single- and double-ended readout. Since significant advances in solid angle collection, fill factor, and quantum efficiency are possible, the plots of CRT span the range from 5% to 100% photon conversion factor. The timing jitter for the photodetector is described by a Gaussian distribution with the fwhm parameter *J*.

### 5.1. Scintillator and photodetector parameters used in the calculations

Table 2 lists the properties of the three scintillators used in the single-ended and double-ended CRT calculations. The first two are in common use, and the third could be based on allowed donor-acceptor radiative transitions in a heavy-atom semiconductor (Lehmann 1966, Bourret-Courchesne *et al* 2009, Derenzo *et al* 2013).

The optical photon timing dispersion was calculated from equations (2*a*) and (2*b*) using *d*_1_ = 0.008 73 ns and *d*_2_ = 0.0186 ns cm^−1^. We simulated a high-performance photodetector close-coupled to a high-bandwidth amplifier whose SER is described by a bi-exponential with 0.2 ns rise time and 2 ns decay time (equation (5)), unless otherwise noted. In appendix B we show that increasing the SER by a factor of five to 1 ns rise time and 10 ns decay time changes the optimal trigger fraction but has little effect on the coincidence response time. The insensitivity to SER shape was also shown in (Derenzo *et al* 2014).

### 5.2. Results for single-ended readout without DOI information

**Figure 9:**
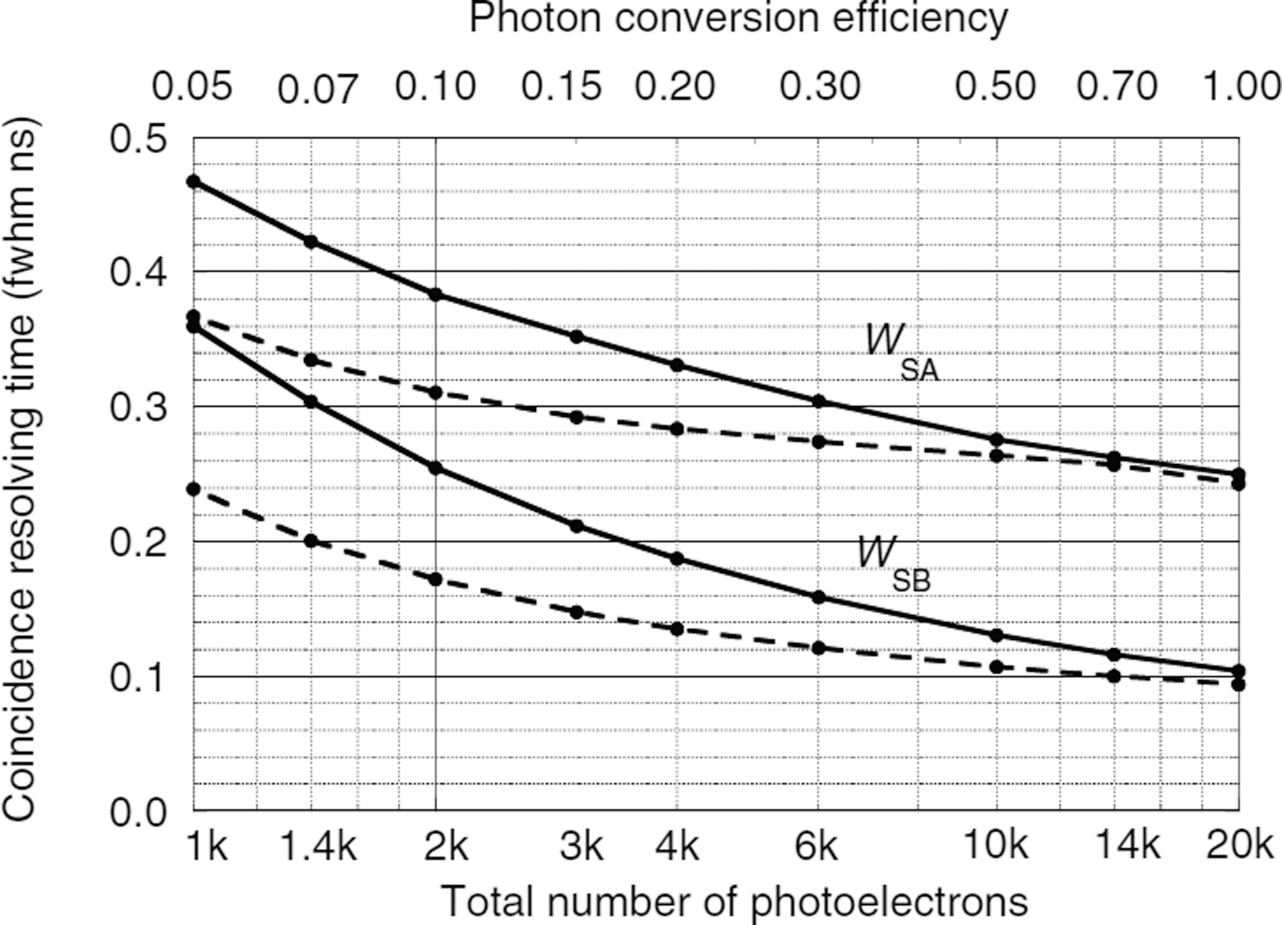
CRTs *W*_SA_ and *W*_SB_ as a function of the number of photoelectrons using single-ended photodetector readout of two LSO crystals. Photodetectors are either on surface A or surface B with the opposite surface reflective. Solid lines for a photodetector time jitter *J* = 0.4 ns fwhm. Dashed lines for *J* = 0.0 ns fwhm.

**Figure 10:**
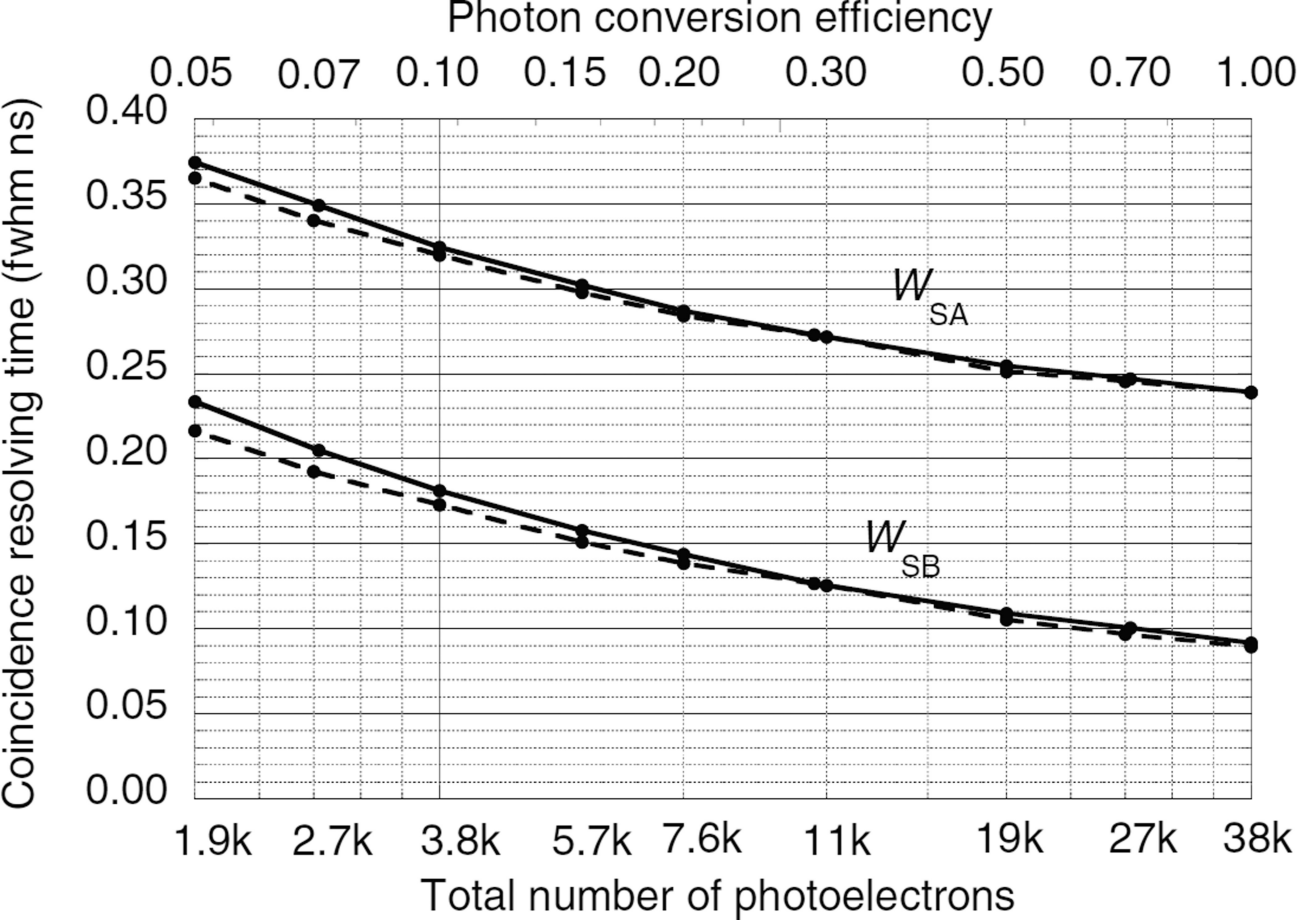
CRTs *W*_SA_ and *W*_SB_ as a function of the number of photoelectrons using single-ended photodetector readout of two LaBr_3_ : Ce crystals. Photodetectors are either on surface A or surface B with the opposite surface reflective. Solid lines for a photodetector time jitter *J* = 0.4 ns fwhm. Dashed lines for *J* = 0.0 ns fwhm.

**Figure 11:**
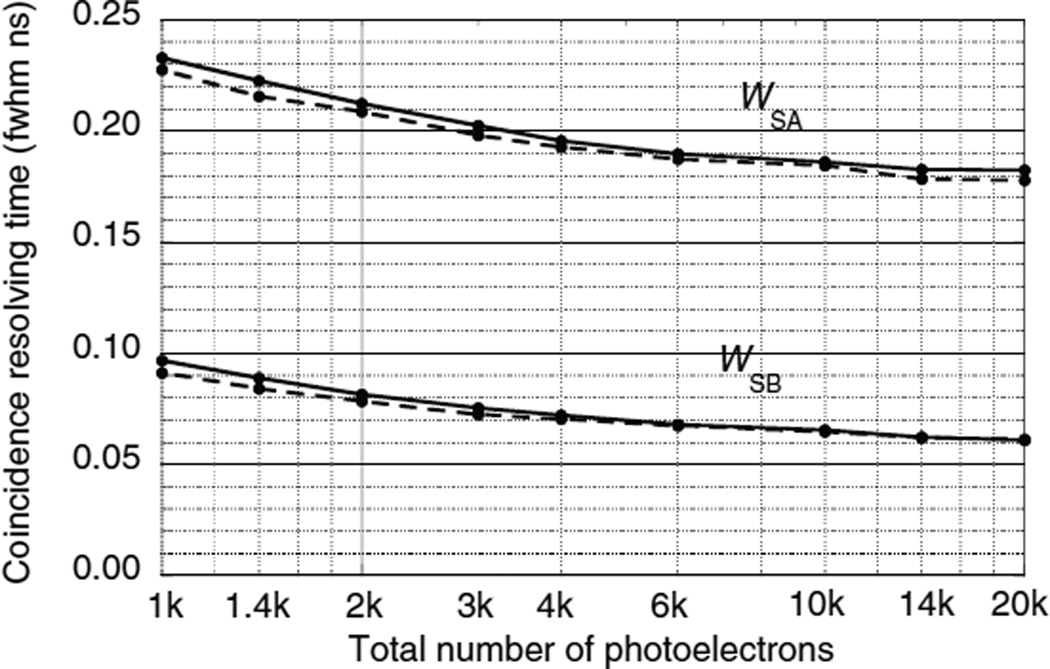
CRTs *W*_SA_ and *W*_SB_ as a function of the number of photoelectrons using single-ended photodetector readout of two hypothetical ultra-fast scintillators. Photodetectors are either on surface A or surface B with the opposite surface reflective. Solid lines for a photodetector time jitter *J* = 0.4 ns fwhm. Dashed lines for *J* = 0.0 ns fwhm.

Figures 9–11 show the CRT as a function of the number of photoelectrons for the single-ended readout of surface A or surface B with the opposing surfaces reflective. These were calculated using the Monte Carlo procedures described in sections 4.1–4.3. Note that the scales on the vertical axes are different. In all three figures the CRT for the photodetector on the B surface are considerably lower than the detector on the A surface. The reasons for this are explained below.

In the case where the photodetector is on the B surface the photodetector receives two swarms of optical photons. After the annihilation photon enters surface A the leading edge of the first swarm arrives at surface B at time *Z*/*c* + *n*(*L* − *Z*)/*c*. The photons reflected from surface A form a delayed swarm whose leading edge arrives at surface B at time *Z*/*c* + *n*(*L* + *Z*)/*c* (see section 4.3.4). The leading edge of the two swarms are separated by the time 2*nZ*/*c*.

In the case where the photodetector is on the A surface the leading edge of the swarm arrives at time *Z*/*c* + *n*Z/*c*. The leading edge of the delayed swarm reflected from surface B arrives at time *Z*/*c* + *n*(2*L* − *Z*)/*c* (see section 4.2.4). The leading edge of the two swarms are separated by the time 2*n*(*L* − *Z*)/*c*.

The photodetector on surface A has higher CRTs that the photodetector on surface B for two reasons: (1) variations in the DOI Δ*Z* result in larger time variations in the arrival of the leading edge of the direct swarm [Δ*Z*(1 + *n*)/*c* versus Δ*Z*(1−*n*)/*c*], and (2) due to the exponential nature of the DOI distribution, low values of *Z* are more probable and the time difference between the arrival of the two swarms is greater [2*n*(*L* − *Z*)/*c* versus 2*nZ*/*c*] so that the best leading edge trigger fraction is less optimal for both swarms.

For the case of LSO (figure 9) the CRT decreases as the number of photoelectrons *N*_pe_ increases. At low *N*_pe_ the CRT is relatively large and differences due to different values of the photodetector timing jitter *J* can be seen. At large values of *N*_pe_ the CRT is dominated by variations in the DOI and reaches an asymptotic limit. For *N*_pe_ = 10^6^ photoelectrons and *J* = 0.2 ns fwhm, *W*_SA_ = 0.193 and *W*_SB_ = 0.056 ns fwhm.

In contrast, LaBr_3_ : Ce (figure 10) has a shorter decay time, and a higher luminosity than LSO which results in a lower CRT for the same photon conversion efficiency. But it also has a larger attenuation length and index of refraction than LSO which increases the effect of variations in the DOI. For *N*_pe_ = 10^6^ photoelectrons and *J* = 0.2 ns fwhm, *W*_SA_ = 0.207 and *W*_SB_ = 0.061 ns fwhm.

The CRT of the hypothetical ultra-fast scintillator (figure 11) is dominated by variations in the DOI and the dependence on *N*_pe_ is even weaker than the other two scintillators. For *N*_pe_ = 10^6^ photoelectrons and *J* = 0.2 ns fwhm, *W*_SA_ = 0.172 and *W*_SB_ = 0.059 ns fwhm.

### 5.3. Results for double-ended readout

The CRT for double-ended readout is lower than that of single-ended readout because (1) both optical photon swarms reach the photodetectors directly and with minimum delay, (2) the ratio of the photodetector pulse heights can be used to estimate the DOI and correct for annihilation photon, optical photon, and trigger delays.

**Figure 12:**
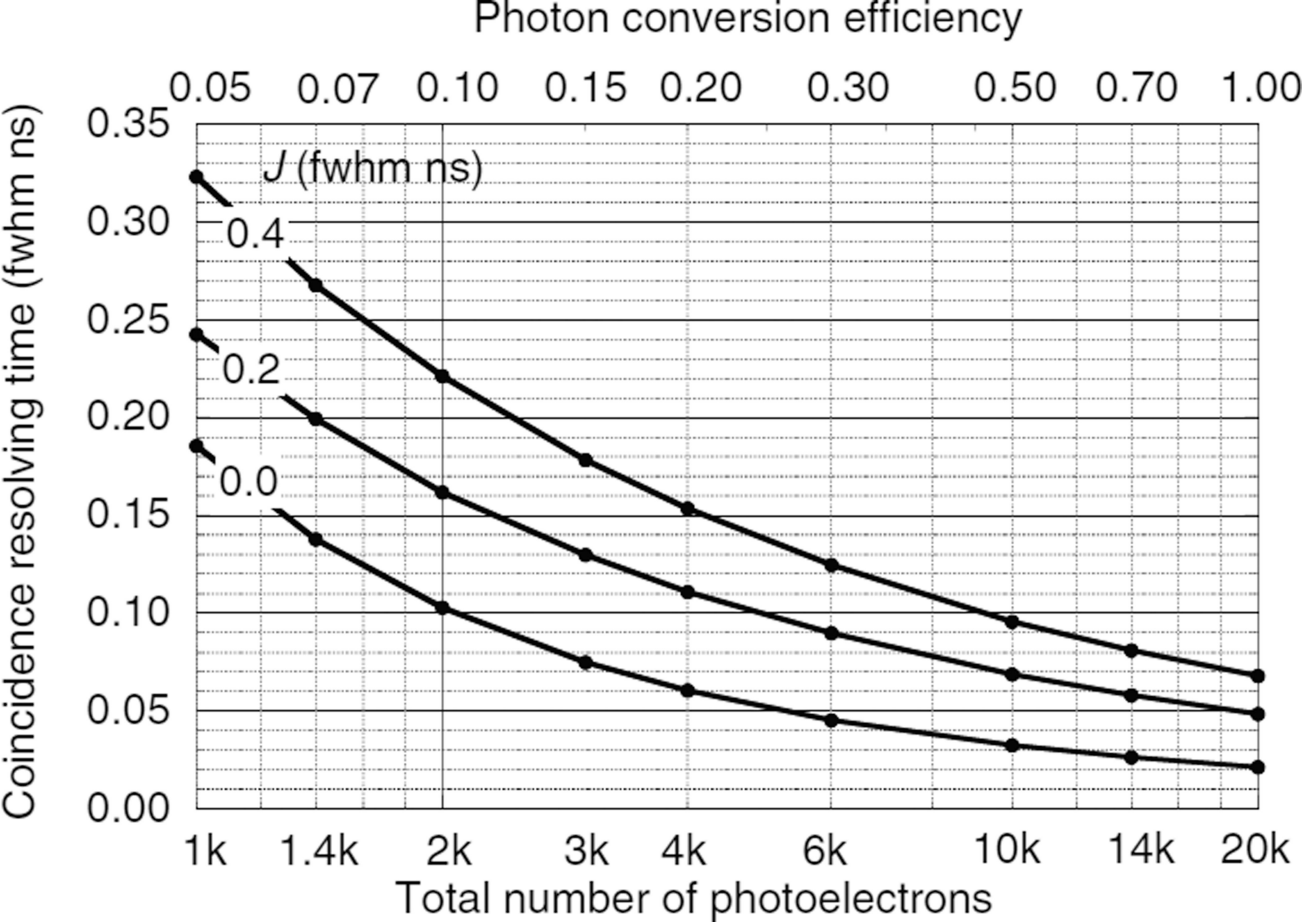
CRT *W*_WAB_ for two LSO scintillators as a function of the total number of photoelectrons and photon conversion efficiency for photodetector transit time jitters *J*.

**Figure 13:**
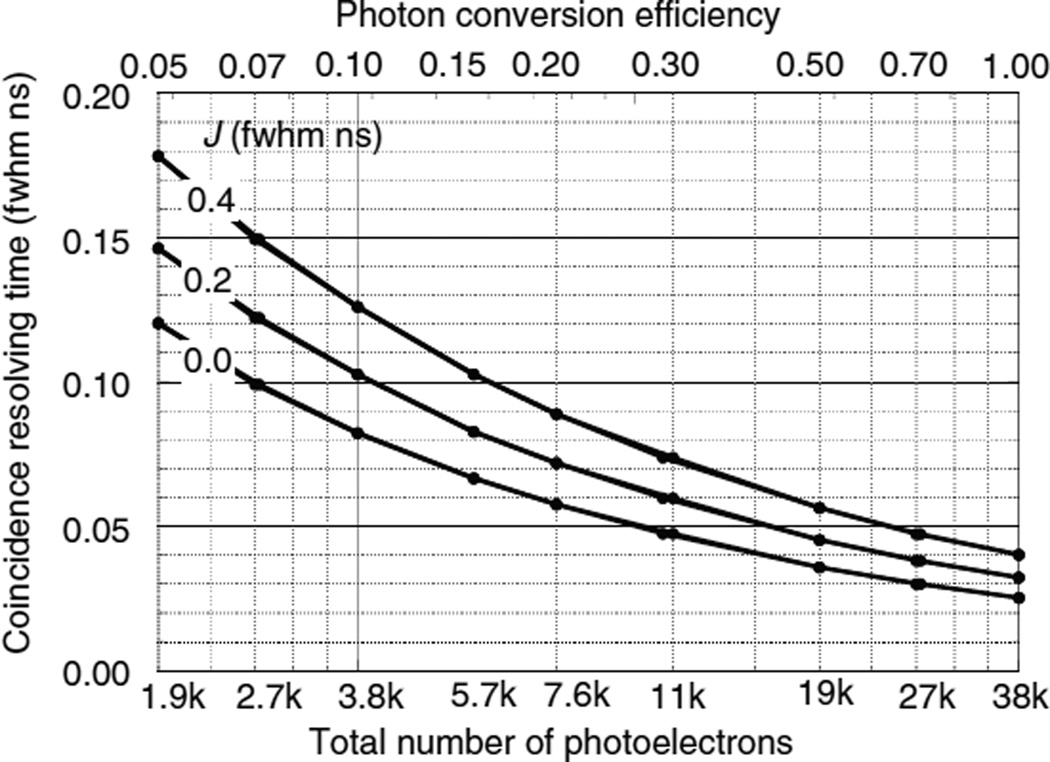
CRT *W*_WAB_ for two LaBr_3_ : Ce scintillators as a function of the total number of photoelectrons and the photon conversion efficiency for photodetector transit time jitters *J*.

**Figure 14:**
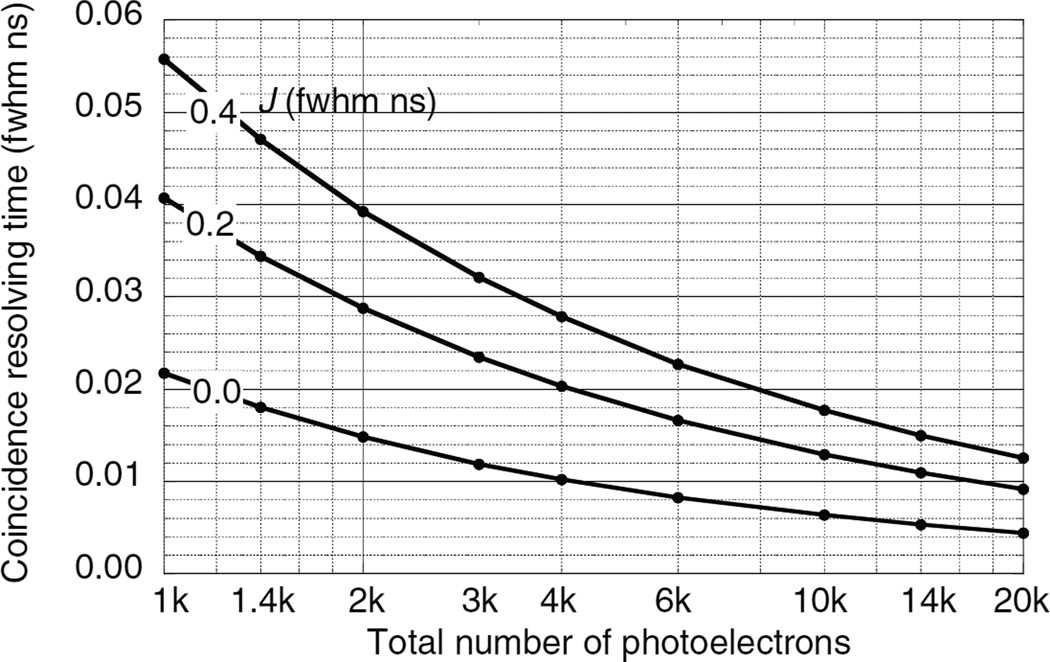
CRT *W*_WAB_ for two hypothetical ultra-fast scintillators as a function of the number of photoelectrons for photodetector transit time jitters *J*.

Tables 3–5 and figures 12–14 show the results of the Monte Carlo calculations using the procedures of section 4.4 for the three scintillators whose properties are listed in table 2. *N*_pe_ is the number of photoelectrons summed in both photodetectors. *J* is the timing jitter of the photodetectors (fwhm ns). *W_Z_* is the uncertainty in the DOI (fwhm cm) using the observed number of photoelectrons *m*_A_ and *m*_B_, and the method in section 4.4.9. For all three scintillators 4000 photoelectrons provide a depth uncertainty *W*_Z_ less than 0.15 cm fwhm, which corresponds to 0.005 ns fwhm at the speed of light and is a minor contribution to the CRT.

*W*_DA_ and *W*_DB_ are the CRT values using only photodetectors A and B, respectively, and optimal trigger fractions. Correcting for the annihilation photon, optical photon, and trigger delays results in two separate estimates for the annihilation photon arrival times *E*_A_ and *E*_B_, and their CRT uncertainties are *W*_EA_ and *W*_EB_. *W*_EAB_ is the fwhm of the simple average of *E*_A_ and *E*_B_. *W*_WAB_ is the CRT for of the inverse variance weighted average of *E*_A_ and *E*_B_.

As shown in appendix D, double-ended readout can estimate the DOI and almost perfectly correct for all depth-dependent factors (annihilation photon, optical photon, and trigger delays). As a result, the tables in (Derenzo *et al* 2014) can be used to scale the *W*_WAB_ values in tables 3–5 to other values of rise times and photodetector timing jitters. For example, for >100 photoelectrons per ns decay time and 0.2 ns photodetector timing jitter, increasing the scintillator rise time from 0 to 0.2 ns increases the timing precision by 31% and 26% for optical dispersion parameters of 0 and 0.1 ns, respectively.

### 5.4. Comparison between constant-fraction and leading edge timing discrimination

Derenzo *et al* (2014) show that the trigger level for optimal timing precision is proportional to the pulse height so we have used constant fraction timing discrimination elsewhere in this paper. However, since simple leading edge discrimination is so much easier to implement the question arises as to how much it degrades the CRT in PET where variations in DOI result in variations in pulse height.

Table 6 shows that for the case of LSO with the properties listed in table 2 and 4000 total photoelectrons in photodetectors with 0.2 ns fwhm timing jitter, leading-edge discrimination provides essentially the same CRT as constant-fraction discrimination. This is not surprising because both methods trigger at similar levels and figure B1 in appendix B shows that the CRT is not a strong function of the trigger fraction near the minimum.

Note that for constant fraction discrimination an ultra-fast laser can be used to measure the variations in trigger times due to the depth-dependent variations in optical photon transport times. For leading edge discrimination the same measurement also includes the variations in trigger times due to depth-dependent variations in pulse height.

### 5.5. Results for single-ended double-layer readout

In this section we compute the CRT for an alternate readout design where two photodetectors are used not to read out the opposite ends of a crystal but instead are used to read out separate front and back crystals that are half the thickness. Table 7 lists the CRT values for single-ended readout (*W*_SB_), where the DOIs are exponentially distributed along the length of 3 cm and 1.5 cm crystals and compares them to the CRT values (*W*_WAB_) for double-ended readout of 3 cm long crystals where the exponentially distributed DOIs are estimated and used to correct the constant fraction trigger times (sections 4.4.9–4.4.14). It is shown in appendix D that the double-ended readout essentially eliminates the effects of the variations in the DOI so it is understandable that the CRTs for single ended readout of 1.5 cm crystals lies between the CRTs for single-ended and double-ended readout of 3 cm crystals.

Since both double-layer and double-ended readout require the same number of photodetectors and support electronics, there are clear advantages in double-ended readout for improving the CRT and providing the ability to estimate the DOI and reduce parallax errors in the reconstructed images. The advantages in improved CRT are especially clear for values below 0.1 ns fwhm.

## 6. PET TOF sensitivity figure of merit

We define a figure of merit for TOF PET (adopted from Derenzo (1982), Conti *et al* (2009)) that is unity for whole-body PET using LSO without TOF information and equal to the sensitivity advantage when TOF information is available.

$$FOM=6.5E^{2}/CRT$$

FOM = 1 for LSO (*E* = 0.6) without TOF and a 35 cm diameter emission region (equivalent to CRT = 2.34 ns fwhm). This formula allows different scintillators to be compared by combining (1) the joint photopeak efficiency *E*^2^ for detecting both annihilation photons and (2) the variance reduction factor (Budinger *et al* 1977, Snyder *et al* 1981, Vunckx *et al* 2010), which is inversely proportional to the CRT. Table 8 compares the FOM for three scintillators with the parameters listed in table 2, using single-ended and double-ended readout by photodetectors with 0.2 ns fwhm jitter.

## 7. Discussion

In this work we explored the limiting factors in the CRT of scintillator pairs read out by photodetectors coupled to one or both opposing surfaces. The annihilation photons interact at different depths in the scintillator, resulting in random variations in the number, transport time, and time dispersion of the optical photons that arrive at the entrance and rear surfaces.

In summary we compared five methods for estimating the time at which annihilation photons enter a scintillation crystal, and these are listed with generally increasing CRT:

1. **Single-ended readout of the entrance surface (*W*_SA_)**. This readout has the worst timing precision because DOI variations Δ*Z* result in timing variations Δ*Z*(*n* + 1)/*c* of the leading edge of the direct photon swarm.
2. **Single-ended readout of the rear surface (*W*_SB_)**. This is preferred relative to (1) because the timing variations in the leading edge of the direct photon swarm are Δ*Z*(*n* − 1)/*c* and the delayed photon swarm reflected from the entrance surface arrives sooner and has a better chance of contributing to the timing information.
  A real-time analysis of the pulse shape for methods (1) or (2) could possibly estimate the DOI and give CRTs similar to *W*_EA_ or *W*_EB_.
3. **Double-ended readout and a simple average of the digitized trigger times (*W*_DAB_)**. This corrects for the depth-dependent variations in the average optical photon delay but not for depth-dependent variations in trigger times due to variations in annihilation photon transport times or optical photon time dispersion. This is the method reported in (Seifert and Schaart 2015).
  The last two methods that follow use double-ended readout where the ratio of the photodetector pulse heights is used to estimate the DOI and correct for depth-dependent annihilation photon, optical photon, and trigger delays. Note that the trigger delay depends not only on the trigger fraction but also on the DOI because the shape of the photodetector pulse depends on the optical photon time dispersion.
4. **Double-ended readout and a simple average of the corrected trigger times (*W*_EAB_)**. Since the relative variance of the corrected trigger times depends strongly on depth, a simple average does not provide the best statistical estimate of the entrance time.
5. **Double-ended readout and a statistically weighted average of the corrected trigger times (*W*_WAB_)**. Correcting for all depth-dependent effects and using the inverse variances as weighting factors results in the best statistical estimate of the entrance time and results in an almost perfect elimination of depth-dependent uncertainties (see appendix D). Unlike single-ended readout, the entrance photodetector A provides better timing information than the rear photodetector B because it receives more photons and the optical photon time dispersion is less.

Method (3) requires a time digitizer for each photodetector and subsequent digital signal processing to compute the average of the two trigger times. With amplitude digitizers and some additional signal processing, methods (4) and (5) provide more accurate CRT values as well as the DOI information needed to correct for parallax error in the reconstructed images.

## 8. Conclusions

- In the absence of DOI information, the CRT fwhm (*W*_SB_) for single-ended readout of the rear surfaces of 3 mm × 3 mm × 30 mm crystals is limited to about 0.15 ns for both LSO and LaBr_3_ : Ce, and 0.070 ns for a hypothetical ultra-fast scintillator with comparable luminosity, zero rise time, and 1 ns decay time.
- The CRT for single-ended readout reaches a plateau at high values of *N*_pe_ because of the contributions from depth-dependent variations the transport time and diffusion of the optical photons, which are not known on an event-by-event basis.
- Double-ended readout allows for an accurate estimation of the DOI and correction for the depth-dependent annihilation photon, optical photon, and trigger delays. This improves the CRT fwhm (*W*_WAB_) to about 0.09 ns for LSO, 0.07 ns for LaBr_3_ : Ce, and below 0.02 ns for the hypothetical ultra-fast scintillator.
- Double-ended readout with constant-fraction discrimination and correction for the depth-dependent annihilation photon, optical photon, and trigger delays allows almost perfect elimination of depth-dependent uncertainties and the CRT (*W*_WAB_) is only about 10% higher than the statistical lower bound. This shows that constant-fraction discrimination averages over the times of the most useful photoelectrons in a statistically efficient manner.
- In double-ended readout the photodetector at entrance photodetector A provides more timing information than the photodetector at rear surface B. This is different than single-ended readout, which is best with the photodetector at the rear surface.
- When using double-ended readout the simple average of the digitized trigger times compensates for depth-dependent variations in the optical photon delay but does not correct for variations in the annihilation photon and trigger delays. (This yields the CRT *W*_DAB_)
- With correction for depth-dependent variations in pulse height, simple leading edge discrimination performs as well as constant fraction discrimination.
- While there are opportunities for improving the number of photoelectrons and the photodetector timing jitter, reducing the scintillator decay time (to increase the number of photoelectrons per ns decay) will have the largest impact in reducing the CRT.

## Figures and Tables

**Table 1:** Comparison between measurements and lower bound calculations from reference (Seifert *et al* 2012b)and the Monte Carlo calculations from this work

	Lu_2_SiO_5_ : Ce	LaBr_3_ : Ce
Attenuation length μ (cm)	1.2	2.3
Crystal length (cm)	0.5	0.5
Rise time τ_r_ (ns)	0.09	0.4
Decay time τ_d_ (ns)	43.8	15
Index of refraction *n*	1.82	2.1
Total number of photoelectrons *N*_pe_	4700	6200
Photodetector SER timing jitter *J* (ns fwhm)	0.3	0.3
Measured CRT (ns fwhm) (Seifert *et al* 2012b)	0.138	0.095
Statistical lower bound (ns fwhm) (Seifert *et al* 2012b)	0.140	0.095
Calculated CRT (ns fwhm) (section 4.3 this work)	0.138	0.093

**Table 2:** Model parameters for Monte Carlo calculations of the CRT for three scintillators.

	LSO	LaBr_3_ : Ce	Ultra-fast
Size (mm)	3 × 3 × 30	3 × 3 × 30	3 × 3 × 30
Attenuation length μ for 511 keV photons (cm)	1.2	2.3	1.2a
Index of refraction *n*	1.82	2.1	2^a^
Photons per 511 keV *N*_pe_	20 000	38 000	20 000^a^
Rise time τ_r_ (ns)	0.0	0.2 (Glodo *et al* 2005)	0.0^a^
Decay time τ_d_ (ns)	37	18 (Glodo *et al* 2005)	1^a^

a Hypothetical values.

**Table 3:** Double-ended readout for two Lu_2_SiO_5_ : Ce, Ca scintillators. See appendix A for parameter definitions. The last seven columns are CRT values in ns fwhm for optimal trigger level fractions.

*N*_pe_	*J* (fwhm)	*W*_Z_ (cm)	*W*_DA_	*W*_DB_	*W*_DAB_	*W*_EA_	*W*_EB_	*W*_EAB_	*W*_WAB_
1k	0.0	0.26	0.400	0.390	0.230	0.257	0.360	0.220	0.186
2k	0.0	0.19	0.320	0.241	0.146	0.144	0.206	0.124	0.103
4k	0.0	0.13	0.283	0.164	0.109	0.086	0.124	0.075	0.061
10k	0.0	0.08	0.261	0.116	0.091	0.047	0.068	0.041	0.032
20k	0.0	0.06	0.251	0.097	0.086	0.030	0.045	0.027	0.021
1k	0.2	0.26	0.448	0.444	0.275	0.327	0.419	0.264	0.242
2k	0.2	0.19	0.366	0.303	0.191	0.218	0.272	0.173	0.162
4k	0.2	0.13	0.326	0.224	0.143	0.149	0.184	0.118	0.111
10k	0.2	0.09	0.299	0.167	0.110	0.093	0.114	0.073	0.069
20k	0.2	0.06	0.289	0.143	0.097	0.065	0.080	0.051	0.048
1k	0.4	0.26	0.534	0.550	0.350	0.435	0.529	0.341	0.323
2k	0.4	0.19	0.426	0.387	0.246	0.297	0.358	0.232	0.221
4k	0.4	0.13	0.363	0.284	0.179	0.206	0.248	0.161	0.153
10k	0.4	0.08	0.322	0.204	0.129	0.129	0.154	0.100	0.096
20k	0.4	0.06	0.307	0.170	0.108	0.091	0.109	0.071	0.068

**Table 4:** Double-ended readout for two LaBr_3_ : Ce scintillators. See appendix A for parameter definitions. The last last seven columns are CRT values in ns fwhm for optimal trigger level fractions.

*N*_pe_	*J* (fwhm)	*W*_Z_ (cm)	*W*_DA_	*W*_DB_	*W*_DAB_	*W*_EA_	*W*_EB_	*W*_EAB_	*W*_WAB_
1.9k	0.0	0.19	0.386	0.257	0.158	0.172	0.196	0.130	0.120
3.8k	0.0	0.14	0.355	0.206	0.127	0.118	0.135	0.089	0.082
7.6k	0.0	0.10	0.336	0.174	0.111	0.083	0.094	0.062	0.058
19k	0.0	0.06	0.320	0.149	0.100	0.052	0.059	0.039	0.036
38k	0.0	0.04	0.312	0.136	0.096	0.036	0.041	0.027	0.025
1.9k	0.2	0.19	0.415	0.293	0.179	0.208	0.231	0.154	0.146
3.8k	0.2	0.14	0.382	0.237	0.141	0.145	0.161	0.108	0.103
7.6k	0.2	0.10	0.362	0.203	0.118	0.102	0.113	0.076	0.072
19k	0.2	0.06	0.347	0.178	0.103	0.064	0.071	0.047	0.045
38k	0.2	0.04	0.340	0.166	0.097	0.046	0.050	0.034	0.032
1.9k	0.4	0.19	0.448	0.336	0.207	0.252	0.277	0.186	0.178
3.8k	0.4	0.14	0.406	0.269	0.159	0.177	0.195	0.131	0.126
7.6k	0.4	0.10	0.382	0.228	0.129	0.125	0.137	0.092	0.089
19k	0.4	0.06	0.364	0.197	0.108	0.079	0.087	0.058	0.056
38k	0.4	0.04	0.356	0.184	0.100	0.056	0.062	0.042	0.040

**Table 5:** Double-ended readout for two hypothetical ultra-fast scintillators. See appendix A for parameter definitions. The last seven columns are CRT values in ns fwhm for optimal trigger level fractions.

*N*_pe_	J (fwhm)	*W*_Z_ (cm)	*W*_DA_	*W*_DB_	*W*_DAB_	*W*_EA_	*W*_EB_	W_EAB_	W_WAB_
1k	0.0	0.26	0.261	0.102	0.084	0.044	0.036	0.023	0.022
2k	0.0	0.19	0.258	0.095	0.083	0.031	0.024	0.016	0.015
4k	0.0	0.13	0.255	0.092	0.083	0.022	0.017	0.011	0.010
10k	0.0	0.08	0.252	0.088	0.082	0.014	0.010	0.007	0.006
20k	0.0	0.06	0.251	0.086	0.081	0.010	0.007	0.005	0.004
1k	0.2	0.26	0.298	0.146	0.092	0.066	0.066	0.042	0.041
2k	0.2	0.19	0.293	0.136	0.087	0.047	0.047	0.029	0.029
4k	0.2	0.13	0.288	0.129	0.085	0.033	0.033	0.021	0.020
10k	0.2	0.08	0.285	0.123	0.083	0.021	0.021	0.013	0.013
20k	0.2	0.06	0.282	0.119	0.081	0.015	0.015	0.009	0.009
1k	0.4	0.26	0.317	0.171	0.099	0.084	0.089	0.057	0.056
2k	0.4	0.19	0.310	0.157	0.091	0.060	0.063	0.040	0.039
4k	0.4	0.13	0.306	0.148	0.086	0.042	0.045	0.028	0.028
10k	0.4	0.08	0.301	0.140	0.083	0.027	0.028	0.018	0.018
20k	0.4	0.06	0.297	0.136	0.081	0.019	0.020	0.013	0.013

**Table 6:** Comparison between constant fraction and leading edge timing discrimination for a total *N*_pe_ = 4000 photoelectrons and photodetector timing jitters of 0.2 ns fwhm.

Discrimination type	Trigger level	*W*_DAB_	*W*_EAB_	*W*_WAB_
Optimal constant fraction	0.018	0.143	0.118	0.111
Optimal leading edge	2.0^a^	0.140	0.120	0.112

a For front photodetector A this corresponds to a trigger fraction of 0.014 of the average amplitude 140.5 SER. For rear photodetector B this corresponds to a trigger fraction of 0.019 of the average amplitude 104.3 SER.

**Table 7:** Comparison of CRT values (ns fwhm) for single-ended readout of 3 and 1.5 cm deep crystals (*W*_SB_) and for double ended readout of 3 cm deep crystals (*W*_WAB_).

τ_r_ (ns)	τ_d_ (ns)	*J* (ns fwhm)	*N*_pe_	*W*_SB_ (*L* = 3 cm)	*W*_SB_ (*L* = 1.5 cm)	*W*_WAB_ (*L* = 3 cm)
0	30	0.2	1000	0.278	0.227	0.216
0	30	0.2	10 000	0.128	0.085	0.062
0	30	0.4	1000	0.328	0.292	0.288
0	30	0.4	10 000	0.134	0.098	0.087
0.2	30	0.2	1000	0.324	0.279	0.271
0.2	30	0.2	10 000	0.134	0.096	0.081
0.2	30	0.4	1000	0.361	0.330	0.329
0.2	30	0.4	10 000	0.140	0.109	0.099
0	1	0.2	1000	0.097	0.059	0.040
0	1	0.2	10 000	0.066	0.035	0.013
0	1	0.4	1000	0.101	0.067	0.055
0	1	0.4	10 000	0.066	0.035	0.018
0.2	1	0.2	1000	0.102	0.066	0.052
0.2	1	0.2	10 000	0.062	0.036	0.016
0.2	1	0.4	1000	0.104	0.073	0.063
0.2	1	0.4	10 000	0.068	0.037	0.020

**Table 8:** Comparison of the FOM for three scintillators using single-ended and double-ended readout.

	LSO	LaBr_3_ : Ce	Ultra-fast
Full energy detection efficiency	0.6^a^	0.4^a^	0.5^b^
Total number of photoelectrons* N*_pe_	4000	7400	4000^b^
Single-ended surface B CRT *W*_SB_ (ns fwhm)	0.16	0.14	0.070^b^
Single-ended surface B figure of merit (FOM)^1^	14.6	7.4	23^b^
Double-ended CRT *W*_WAB_ (ns fwhm)	0.111	0.072	0.020^b^
Double-ended figure of merit (FOM)^2^	21.0	14.4	81b

a Typical values for purposes of comparison.

b Hypothetical values.

## Appendix A. Variables and abbreviations

Table A1 lists the variables used in the calculations, and table A2 lists the abbreviations used in the text.

**Table A1:** Glossary of variables used in the calculations. All times in ns. All distances in cm.

*a*	Fraction of light received by a photodetector when the interaction point is at the same end of the scintillator and the opposite end is absorbing (equations (1*a*) and (1*b*))
*c*	Speed of light in a vacuum (29.979 cm ns^−1^)
*D*_Ak_	Optical photon time dispersion parameter from photons emitted at depth *Z_k_* and either absorbed at rear surface B after being reflected at entrance surface A (equation (8*b*)), or absorbed directly at entrance surface A (equations (6*a*) and (9*a*))
*D*_Bk_	Optical photon time dispersion parameter from photons emitted at depth *Z_k_* and either absorbed at entrance surface A after being reflected at rear surface B (equation (6*b*)) or absorbed directly at rear surface B (equations (8*a*) and (9*b*))
*d*_1_	Constant coefficient for optical photon time dispersion (equations (2*a*) and (2*b*))
*d*_2_	Quadratic coefficient for optical photon time dispersion (equations (2*a*) and (2*b*))
δ_Ajn_	Trigger delay from the arrival of the first possible photon at photodetector A (from an interaction at *Z_j_*) to the trigger time *T*_Akn_.
δ_Bjn_	Trigger delay from the arrival of the first possible photon at photodetector B (from an interaction at *Z_j_*) to the trigger time *T*_Bkn_.
*E*_A*kn*_	Estimate of the entrance time of the annihilation photon using *T*_Akn_ and correcting for the depth-dependent annihilation photon, optical photon, and trigger delays (equation (11*a*)) (should be zero with perfect timing)
*E*_B*kn*_	Estimate of the entrance time of the annihilation photon using *T*_Bkn_ and correcting for the depth-dependent annihilation photon, optical photon, and trigger delays (equation (11*b*)) (should be zero with perfect timing)
*E*_AB*kn*_	Simple average of the corrected entrance times *E*_Akn_ and *E*_Bkn_ for double-ended read-out (equation (12))
*E*_WAB*kn*_	Inverse-variance weighted average of *E*_Akn_ and *E*_Bkn_ (equation (13))
*f*_A_(*Z*)	Fraction of photons that reach surface A directly from an ionization event at depth *Z* (equation (1*a*))
*f*_B_(*Z*)	Fraction of photons that reach surface B directly from an ionization event at depth *Z* (equation (1*b*))
*F_n_*	Fractional trigger level (tabulated from 0 to 1 on index *n*)
*F*_opt_	Fractional trigger level for minimum CRT (fraction of peak amplitude *P*) (figure B1)
*H*	Number of photoelectrons contributing to the photodetector output pulse at the trigger time (table B1)
*J*	Single photoelectron time jitter of the photodetector (Gaussian fwhm)
*L*	Length of scintillator (distance between surfaces A and B)
μ	Attenuation length for 511 keV annihilation photons in the scintillator
*m*_A*k*_	Observed number of photoelectrons in photodetector A from an interaction at depth *Z_k_* (randomly distributed with mean = *N*_Ak_, variance = *N*_Ak_) (equation (4*a*))
*m*_B*k*_	Observed number of photoelectrons in photodetector B from an interaction at depth *Z_k_* (randomly distributed with mean = *N*_Bk_, variance = *N*_Bk_) (equation (4*b*))
*n*	Index of refraction of the scintillator at the wavelength of the scintillation light
*N*_g_	Number of ionization events used in each Monte Carlo calculation
*N*_pe_	Number of photoelectrons produced in the photodetector (single-ended readout) or the sum in both photodetectors (double-ended readout). This is the product of the scintillator luminosity (photons/MeV), the energy deposited (511 keV), the photon transport efficiency, the photodetector fill factor, and the photodetector quantum efficiency.
*N*_A*k*_	Expected number of photoelectrons in photodetector A for an interaction at depth *Zk* (equation (3*a*)).
*N*_B*k*_	Expected number of photoelectrons in photodetector B for an interaction at depth *Z_k_* (equation (3*b*)).
*P*_A*k*_(max)	Maximum amplitude of the photodetector A output pulse for interaction *k*
*P*_B*k*_(max)	Maximum amplitude of the photodetector B output pulse for interaction *k*
*P*(*t*)	Photodetector output pulse amplitude at time *t* summed over all earlier photoelectron responses (equation (7))
*R_m_*	Random number from a set uniformly distributed between 0 and 1
*S*(*t*)	Photodetector SER (equation (5))
*S*_r_	Rise time of SER bi-exponential (equation (5))
*S*_d_	Decay time of SER bi-exponential (equation (5))
τ_d_	Decay time of the scintillator
τ_r_	Rise time of the scintillator
*T*_A*kn*_	Trigger time of photodetector A for interaction *k* at trigger level *F_n_* P_A*k*_(max) (sections 4.2.7, 4.3.7, and 4.4.7)
*T*_B*kn*_	Trigger time of photodetector B for interaction *k* at trigger level *F_n_* P_B*k*_(max) (sections 4.2.7, 4.3.7, and 4.4.7)
*T*_AB*kn*_	Simple average of *T*_Akn_ and *T*_Bkn_ for double-ended readout. This corrects for the depth-dependent variation in optical photon delay but does not correct for the variations in annihilation photon or trigger delay. (section 4.4.8)
*T_m_*	Creation time of the photodetector output pulse from photoelectron *m* after the arrival of the annihilation photon at the entrance surface (sections 4.2.5, 4.3.5, 4.4.5).
*V*_A*jn*_	Variance in the corrected trigger times *E*_Akn_ averaged over bands of *Z_j_*
*V*_B*jn*_	Variance in the corrected trigger times *E*_Bkn_ averaged over bands of *Z_j_*
*W*_SA_	CRT for single-sided readout with the photodetector at surface A. Calculated as 1.4142 times the fwhm of the distribution of *T*_Akn_ at the optimal trigger fraction (section 4.2.8).
*W*_SB_	CRT for single-sided readout with the photodetector at surface B. Calculated as 1.4142 times the fwhm of the distribution of *T*_Bkn_ at the optimal trigger fraction.
*W*_DAB_	CRT for double-ended readout using a simple average of the *T*_A*kn*_ and *T*_B*kn*_ for each interaction *k* and trigger fraction *F_n_*. Calculated as 1.4142 times the fwhm of the distribution of *T*_ABkn_ at the optimal trigger fraction (section 4.4.13).
*W*_EA_	CRT for double-ended readout using the optimal trigger time of only photodetector A but correcting for the estimated depth *Ẑ* of the distribution of entrance time estimates. Calculated as 1.4142 times the fwhm of the distribution of *E*_Akn_ at the optimal trigger fraction (section 4.4.13) (should be zero with perfect timing).
*W*_EB_	CRT for double-ended readout using the optimal trigger time of only photodetector B but correcting for the estimated depth *Ẑ* of the distribution of entrance time estimates. Calculated as 1.4142 times the fwhm of the distribution of *E*_Bkn_ at the optimal trigger fraction (section 4.4.13) (should be zero with perfect timing).
*W*_EAB_	CRT for double-ended readout using a simple average of *E*_Akn_ and *E*_Bkn_. Calculated as 1.4142 times the fwhm of the distribution of *E*_ABkn_ at the optimal trigger fraction (section 4.4.13).
*W*_WAB_	CRT for double-ended readout using the inverse weighted average of *E*_Akn_ and *E*_Bkn_. Calculated as 1.4142 times the fwhm of the distribution of *E*_WABkn_ at the optimal trigger fraction (section 4.4.13).
*W*_Z_	fwhm of the distribution of differences between Monte Carlo depth *Z* and estimated depth *Ẑ* (section 4.4.15)
*Z_k_*	Depth of interaction for interaction *k*. Randomly distributed according to exponential attenuation (section 4.1.1)
*Ẑ*	Depth of interaction estimated from observed photodetector photoelectron counts *m*_Ak_ and *m*_Bk_ (equation (10))

**Table A2:** Glossary of abbreviations used.

BGO	Bi_4_Ge_3_O_12_ scintillator
CRT	Coincidence response time (fwhm ns)
DOI	Depth of interaction
FOM	Figure of merit (effective sensitivity relative to LSO without TOF)
fwhm	Full-width at half-maximum
LSO	Lu_2_SiO_5_ : Ce scintillator
PET	Positron emission tomography
TOF	Time of flight difference between two annihilation photons
SER	Single electron time response of the photodetector

## Appendix B. Examples of trigger fraction optimization

Figure B1 shows the CRT versus trigger fraction for four different analysis methods and two different SER rise and decay times for a 3 mm × 3 mm × 30 mm LSO scintillator and a total of 4000 photoelectrons. For single-ended readout (*W*_SB_) the 4000 photoelectrons are generated in the photodetector at rear surface B. For double-ended readout a combined sum of 4000 photoelectrons is generated in the two photodetectors. Figure B1(a) is for an SER rise time of 0.2 ns and a decay time of 2 ns. Figure B1(b) is for an SER rise time of 1 ns and a decay time of 10 ns. The latter is apparently a better weighted average of the individual photoelectron pulses and the CRT curves have slightly lower minima. The dots show the trigger fraction values *F_n_* that were used in section 5.

The minimum CRT values in figure B1 require optimizing the trigger fraction to an accuracy of about 30%. This can be done by using a point-like positron source at the center of the tomograph and adjusting the trigger fraction for the minimum CRT. While the optimal trigger fraction does not average the photoelectron times in the best way, the result in the cases published is less than 20% larger than the Cramér-Rao lower bound (Seifert *et al* 2012a, Derenzo *et al* 2014, Gundacker *et al* 2015). Appendix D shows that for a variety of cases the statistically weighted sum of corrected constant-fraction trigger times yields a CRT (*W*_WAB_) that is only about 10% higher than the lower bound.

**Figure B1.** Coincidence resolving time versus trigger fraction for pairs of LSO crystals with 4000 photoelectrons and 0.2 ns fwhm photodetector timing jitter. (a) SER rise time 0.2 ns, decay time 2 ns, (b) SER rise time 1 ns, decay time 10 ns. The dots are located at the trigger fractions *F_n_* used in sections 4.2.7 and 4.4.7.

Table B1 lists results for the three example scintillators described in table 2 and for each photodetector. *N*_pe_ is the average number of photoelectrons. *P* is the average pulse height in units of the maximum amplitude of the SER. *F*_opt_ is the optimal trigger fraction. *P F*_opt_ is the average trigger level. *H* is the number of photoelectrons that contribute to the trigger. *W*_WAB_ is the CRT for the inverse variance weighted average of the corrected trigger times *E*_A_ and *E*_B_. Note that the pulse height *P* divided by the number of photoelectrons *N*_pe_ and the optimal trigger fraction do not depend on *N*_pe_.

**Table B1:** Results for three example scintillators described in table 2 and photodetector timing jitter 0.2 ns fwhm. See text and table A1 for variable definitions.

	LSO	LSO	LSO^a^	LaBr_3_ : Ce	LaBr_3_ : Ce	Ultra- fast	Ultra- fast
*N*_pe_ (total)	1000	4000	4000	1900	7600	1000	4000
Average *N*_pe_ (photodetector A)	575	2302	2302	1030	4120	575	2301
Average *P* (photodetector A)	36.9	140.5	357	115.8	452.8	364.9	1457
Average *N*_pe_ (photodetector B)	424	1697	1697	869	3479	424	1698
Average *P* (photodetector B)	27.7	104.3	263	98.2	383.0	268.8	1075
*F*_opt_	0.018	0.018	0.0024	0.022	0.022	0.065	0.065
*P F*_opt_ (photodetector A)	0.66	2.5	0.86	2.5	10.0	23.7	95
*P F*_opt_ (photodetector B)	0.50	1.9	0.63	2.3	8.4	17.4	70
Average *H*_A_ (photodetector A)	2.1	6.9	8.3	6.1	22	59	235
Average *H*_B_ (photodetector B)	1.7	5.1	6.0	5.2	19	43	169
*W*_WAB_ (ns fwhm)	0.242	0.111	0.109	0.146	0.072	0.041	0.020

a SER rise time 1 ns and decay time 10 ns used in figure B1(b). All other columns had SER rise time 0.2 ns and decay time 2 ns.

## Appendix C. Example of inverse variance weighting factors

Figure C1 shows the inverse variance weighting factors (section 4.4.12) used in the calculation of *W*_WAB_. Unlike single-ended readout, photodetector A provides better timing information than photodetector B because it receives a larger fraction of the photons and the optical photon time dispersion is less. The relative variances of *E*_A_ and *E*_B_ are a strong function of the DOI, and the simple average is not the best statistical estimator of the annihilation photon entrance time.

**Figure C1.** Inverse variance weighting factors (section 4.4.12) for the depth-corrected trigger times *E*_A_ and *E*_B_ for photodetectors A and B coupled to opposing ends of a 3 mm × 3 mm × 30 mm LSO crystal with a combined total *N*_pe_ = 4000 photoelectrons and photodetector jitter times of 0.2 ns fwhm.

## Appendix D. Comparison between CRT values for variable DOI, fixed DOI and the statistical lower bound

Table D1 compares the CRT values for exponentially distributed DOI, fixed DOI and the statistical lower bound for 0.3 cm × 0.3 cm × 3.0 cm crystals with index of refraction = 2, annihilation photon attenuation depth = 1.5 cm and different rise times, decay times, and photodetector timing jitters.

*W*_WAB_ was computed using the method listed in section 4.4 where the DOI was distributed exponentially along the 3 cm length of the crystal according to the annihilation photon attenuation length.

**Table D1:** Comparison between CRTs for variable DOI, fixed DOI and the statistical lower bound. See text for dsetails.

τ_r_ (ns)	τ_d_ (ns)	*J* (ns fwhm)	*N*_pe_	*W*_WAB_^a^	*W*_WAB_ (*Z* ≡ 1.5 cm)^b^	*W*_LB_ (*Z* ≡ 1.5 cm)^b^
0	30	0.2	1000	0.216	0.217	0.199
0	30	0.2	10 000	0.062	0.063	0.060
0	30	0.4	1000	0.288	0.289	0.265
0	30	0.4	10 000	0.087	0.087	0.081
0.2	30	0.2	1000	0.271	0.272	0.247
0.2	30	0.2	10 000	0.081	0.081	0.075
0.2	30	0.4	1000	0.329	0.325	0.298
0.2	30	0.4	10 000	0.099	0.099	0.092
0	1	0.2	1000	0.040	0.039	0.036
0	1	0.2	10 000	0.013	0.012	0.011
0	1	0.4	1000	0.055	0.054	0.049
0	1	0.4	10 000	0.018	0.017	0.015
0.2	1	0.2	1000	0.052	0.051	0.045
0.2	1	0.2	10 000	0.016	0.016	0.014
0.2	1	0.4	1000	0.063	0.062	0.055
0.2	1	0.4	10 000	0.020	0.020	0.017

a Depth of interaction randomly distributed with exponential attenuation.

b Depth of interaction fixed at center of crystal.

*W*_WAB_ (*Z* ≡ 1.5 cm) was computed as in the previous paragraph but all interactions were placed at the center of the crystal so that the optical photon dispersion was similar to the average of the exponentially distributed case and both photodetectors received similar photon swarms. Since the DOI is fixed these numbers agree with those computed using the methods described in (Derenzo *et al* 2014).

*W*_LB_ (*Z* ≡ 1.5 cm) is the statistical lower bound of the CRT and was computed as follows:

For each scintillator rise and decay time, and photodetector single photoelectron jitter in table D1 several × 10^9^ photoelectron time stamps were generated using the method listed in sections 4.4.1–4.4.5 and these were histogrammed into 0.01 ns bins. The photodetector SER is modeled as a delta function in time.

Seven overlapping splines of the form *a_j_* + *b_j_T* + *c_j_T*^2^ (*j* = 1 to 7) were fit to the logarithm of the photoelectron time stamp histogram to provide a smooth, continuous analytical function of time *T* that described the logarithm of the probability density function (PDF).

*N*_pe_ time stamps were generated using the method listed in sections 4.4.1–4.4.5.

The interaction time was determined from a maximum likelihood fit of the PDF to the time stamps generated in step 3. Specifically, the PDF was shifted in time to maximize the sum of the logarithms of the PDF evaluated at each time stamp.

Steps 3 and 4 were repeated 100 000 times and the lower bound CRT was determined as the product of three factors: the standard deviation of the interaction times, the factor 2.355 (to convert to a fwhm), and the factor 1.414 (to convert from the time jitter of a single detector to the CRT of two detectors).

We conclude that the statistically-based corrections for annihilation and optical photon transport times and trigger delays described in section 4 essentially eliminate the effects of random variations in DOI. Furthermore, the values of *W*_WAB_ average 10% higher than those of *W*_LB_ (*Z* ≡ 1.5 cm) showing that constant-fraction timing discrimination is almost as accurate as more complicated schemes that require the knowledge of the individual photoelectron time stamps.
